# Effects of omega-3 fatty acids on chronic pain: a systematic review and meta-analysis

**DOI:** 10.3389/fmed.2025.1654661

**Published:** 2025-11-05

**Authors:** Lei Xie, Xin Wang, Junyang Chu, Xinyao He, Jingting Bao, Yazhi Xi, Xuelian Wei, Qinghe Zhou

**Affiliations:** ^1^Anesthesia Medicine, Zhejiang Chinese Medical University, Hangzhou, China; ^2^Department of Anesthesia and Pain Medicine, Affiliated Hospital of Jiaxing University, Jiaxing, China; ^3^Department of Nursing, Soochow University, Suzhou, China; ^4^Department of Anesthesiology and Pain Medicine, the Second Affiliated Hospital of Jiaxing University, Jiaxing, China

**Keywords:** omega-3 fatty acids, chronic pain, pain management, systematic review, meta-analysis

## Abstract

**Background:**

Chronic pain afflicts approximately 20% of the global adult population and is frequently undertreated, with available pharmacologic options often associated with significant long-term adverse effects. Although omega-3 fatty acids are known for their anti-inflammatory and immunomodulatory effects, current clinical evidence regarding their efficacy in pain management remains inconclusive.

**Objective:**

To determine how well omega-3 fatty acids reduce chronic pain, and to investigate how factors like disease type, dosage, treatment duration, and study design influence their effectiveness.

**Methods:**

We searched four databases (PubMed, Embase, Cochrane Library, and Web of Science) from inception to 14 February 2025 with no language restrictions. Forty-one randomised controlled trials (RCTs; *n* = 3,759) met predefined criteria. Risk of bias was assessed with RoB 2. Pooled standardised mean differences (SMDs) for pain intensity were obtained through random-effects meta-analyses. Subgroup, sensitivity, and publication-bias analyses were also conducted.

**Results:**

Omega-3 fatty acids showed a moderate, statistically and clinically significant reduction in pain intensity with a standardized mean difference (SMD) of −0.55 (95% CI –0.76 to −0.34; *I*^2^ = 87%). The relief was noticeable at 1 month (SMD = −0.27) and improved by 6 months (SMD = −0.83). Lower doses (≤1.35 g/day) were more effective (SMD = −0.60) compared to higher doses (>1.35 g; SMD = −0.53). The benefits were significant for rheumatoid arthritis, migraine, and other mixed chronic pain conditions, but not for osteoarthritis or mastalgia. There was minimal publication bias according to trim-and-fill adjustment, and leave-one-out tests confirmed robust results.

**Conclusion:**

Omega-3 fatty acid supplementation offers a clinically meaningful and time-dependent reduction in chronic pain, particularly at moderate doses and in certain disease contexts. Standardization of outcome measures, dose optimization, and long-term trials are needed to better define its role in pain management.

**Systematic review registration:**

https://www.crd.york.ac.uk/PROSPERO/view/CRD420251035960, Identifier CRD420251035960.

## 1 Introduction

Data from the 2023 U.S. National Health Interview Survey indicated that 24.3% of adults reported experiencing daily pain during the preceding 3 months, and 8.5% suffered from high-impact chronic pain that substantially limited their ability to participate in work and social activities (1). Globally, an estimated 1.5 billion individuals—approximately one in five of the world’s population—are affected by chronic pain (2). A 2024 systematic review including 148 studies and more than 4.3 million patients with chronic pain reported that approximately one-third exhibited signs of dependence and about 10% developed opioid use disorder with long-term therapy, further complicating management owing to reliance on traditional analgesics (3). These findings underscore the urgent need for safer and more sustainable adjuncts or alternatives to conventional analgesics.

Preclinical evidence suggests several biological pathways by which omega-3 fatty acids may exert analgesic effects. Experimental evidence indicates that eicosapentaenoic acid (EPA) and docosahexaenoic acid (DHA) modulate inflammatory pathways by competing with arachidonic acid metabolism, thereby reducing the production of pro-inflammatory prostaglandin E₂ and leukotriene B₄ (4). In addition, they give rise to specialised pro-resolving mediators, including resolvins, protectins, and maresins, which actively promote the resolution of inflammation (5). Within the nervous system, omega-3 fatty acids have been shown to attenuate central sensitisation and neuroinflammation, at least in part by suppressing microglial activation through the SIRT1–HMGB1–NF-κB pathway (6). Taken together, these findings suggest that omega-3 fatty acids may alleviate pain through both peripheral and central mechanisms. Clinical evidence, however, remains inconsistent. At one end of the spectrum, the large-scale trial followed 19,611 community-dwelling older adults for 5.3 years and found that daily supplementation with 1 g of marine omega-3 fatty acids had no effect on pain prevalence or severity compared with placebo (OR = 0.99; 95% CI, 0.94–1.04) (7). In contrast, a 2024 network meta-analysis of 40 randomized controlled trials (*n* = 6,616) reported that high-dose EPA/DHA supplementation produced the greatest reductions in migraine frequency (SMD = −1.36; 95% CI, −2.32 to −0.39) and severity among all prophylactic interventions evaluated (8). More recent trials have provided additional evidence. A 2025 randomized controlled trial demonstrated that daily supplementation with 2,000 mg of EPA significantly reduced migraine headache days and attack frequency in patients with chronic migraine, accompanied by improvements in quality of life (9). Another randomized trial conducted in 2021 among healthy young men reported that 4 weeks of omega-3 supplementation (3 g/day) significantly reduced muscle soreness 24 h after exercise-induced muscle damage (*p* = 0.034) and attenuated the rise in inflammatory cytokines (10).

In this study, we will conduct a rigorous systematic review and meta-analysis of randomized controlled trials to quantitatively evaluate the efficacy of omega-3 fatty acids in managing chronic pain. This synthesis will provide high-quality evidence to inform clinical practice and guide future research into underlying mechanisms.

## 2 Methods

This systematic review and meta-analysis were carried out in strict accordance with the Cochrane Handbook for Systematic Reviews of Interventions. The study design and reporting were in accordance with the Preferred Reporting Items for Systematic Reviews and Meta-Analyses (PRISMA) guidelines (11), ensuring methodological rigor and transparency. The research protocol was prospectively registered in the International Prospective Register of Systematic Reviews under the registration number CRD420251035960. The research question was formulated using the PICO framework: Population—adults with chronic pain; Intervention—omega-3 fatty acids; Comparator—any control condition; and Outcomes—subjective or objective pain measures defined as primary endpoints.

### 2.1 Eligibility criteria

We included only randomized controlled trials (RCTs) that evaluated the effects of omega-3 fatty acid supplementation in chronic pain conditions. Eligible participants were required to have experienced pain for at least 3 months, and trials had to compare omega-3 supplementation with placebo, usual care, sham product, or an active comparator. Studies were required to report at least one pain-related outcome at any follow-up time point. We excluded non-randomized or quasi-experimental studies, animal studies, abstracts without full data, reviews, editorials, and duplicate publications.

### 2.2 Information sources, search strategy, and selection process

A systematic search was performed in PubMed, Embase, the Cochrane Library, and Web of Science from database inception to February 14, 2025. The search strategy combined terms related to omega-3 fatty acids (“omega-3,” “fish oil,” “EPA,” “DHA,” “polyunsaturated fatty acid,” “Omegaven” etc.), trial filters (“randomized controlled trial,” “clinical trial”), and chronic pain descriptors (“chronic pain,” “persistent pain,” “fibromyalgia,” “headache,” “migraine disorders,” etc.). Equivalent keywords and controlled vocabulary (MeSH in PubMed, Emtree in Embase) were applied as appropriate for each database. No restrictions were imposed on language, publication year, or publication status. The full search strategy is provided in the Supplementary material.

Search results were imported into EndNote X9 (Clarivate Analytics), and duplicates were automatically removed. Two reviewers independently screened titles and abstracts, retrieved potentially eligible full texts, and assessed them against the pre-specified inclusion criteria. Discrepancies were resolved through discussion until consensus was achieved. Reasons for exclusion were documented at the full-text stage. In addition, the reference lists of all included studies, relevant reviews, and prior meta-analyses were manually screened to identify additional eligible citations.

### 2.3 Data collection and data items

Data extraction was independently performed by two reviewers using a standardized collection form. The extracted information was systematically organized into spreadsheets and categorized according to key study characteristics, including author and year of publication, country of origin, study design, study duration or follow-up period, sample size, participant demographics (e.g., age), type of chronic pain condition, exposure (intervention and control groups), detailed dosing regimen for the intervention, and the instruments used for pain assessment.

All studies reporting outcome data as means, mean differences, and standard deviations were eligible for inclusion in the meta-analysis. These values were either directly extracted from the original publications or derived from the available data when necessary. A random-effects model was applied to generate pooled estimates, accounting for anticipated heterogeneity across studies and enhancing the external validity of the findings. This approach yields more conservative effect size estimates, which are particularly appropriate when between-study variability is expected.

### 2.4 Risk of bias and study quality assessment

Two reviewers independently evaluated the methodological quality of the included studies using the Cochrane Risk of Bias tool for randomized trials (RoB 2) (12). The assessment covered the following domains: random sequence generation, allocation concealment, blinding of participants and personnel, blinding of outcome assessment, completeness of outcome data, selective reporting, and other potential sources of bias. Discrepancies between reviewers were resolved through consultation with a third investigator. For each domain, the risk of bias was categorized as “low,” “high,” or “unclear.”

### 2.5 Subgroup analyses

We also conducted a series of pre-specified subgroup analyses in addition to the primary meta-analysis examining the association between omega-3 fatty acid supplementation and chronic pain. These stratifications included disease type, type of fatty acid supplementation, pain assessment scale [e.g., Visual Analogue Scale (VAS), McMaster Universities Osteoarthritis Index (WOMAC), other tools], geographic region (e.g., United States vs. other countries), and control type (placebo vs. active comparator), as illustrated in the corresponding forest plots. Further subgroup analyses were performed based on intervention duration, categorizing studies into short-term (<3 months) and long-term (≥3 months) groups, in accordance with previous meta-analytic frameworks (13). We also stratified trials by daily omega-3 fatty acid dosage (≤1.35 g/day *vs.* >1.35 g/day). This threshold was selected based on prior evidence suggesting a therapeutic range between 1.35 and 2.7 g/day (14). For trials that provided dosages in mg/kg/day, the total daily intake was adjusted based on a standard adult body weight of 70 kg.

### 2.6 Data synthesis and statistical analysis

The meta-analysis was performed using Review Manager (RevMan, version 5.3; Cochrane Collaboration) and Stata (version 15.1; StataCorp). Standardized mean differences (SMDs) were adopted as the primary effect size metric. For each study, the SMD was weighted by the inverse of its variance, and pooled estimates with corresponding 95% confidence intervals (CIs) were subsequently calculated.

SMDs were selected because they allow aggregation of results derived from different assessment instruments across studies (e.g., various pain scales). SMDs were calculated by standardizing the mean difference between intervention and control groups using the pooled standard deviation. An SMD of zero indicates no difference between groups. In this analysis, a negative SMD favors the intervention (indicating pain reduction), whereas a positive SMD favors the control group. According to Cohen’s thresholds, an SMDs >0.8 reflects a large effect, >0.5 a moderate effect, and <0.2 a small effect (13).

For studies that did not provide complete data, a normal distribution was assumed, and the mean and standard deviation were estimated from the reported median and interquartile range (IQR) (15). When a single study reported outcomes for multiple doses of the same supplement, the corresponding SMDs were first pooled within that study to generate a single effect size for the primary analysis. These dose-specific results were subsequently examined in subgroup analyses. The present study utilized meta-regression analysis to investigate the effect of Omega-3 fatty acid intervention duration (1, 2, 3, and 6 months) on analgesic efficacy. The duration of intervention was treated as a continuous moderator to rigorously assess whether analgesic efficacy showed a significant linear trend with increasing duration of intervention.

Statistical heterogeneity was evaluated using Cochran’s *Q* test and the *I*^2^ statistic. Both fixed-effect and random-effects models were generated; however, results from the random-effects model were prioritized when heterogeneity was present. A two-sided *p*-value < 0.05 was considered statistically significant. Publication bias was assessed through the use of a funnel plot, Egger’s test (16), and Begg’s test (17).

## 3 Results

### 3.1 Study selection and characteristics

**Figure 1:**
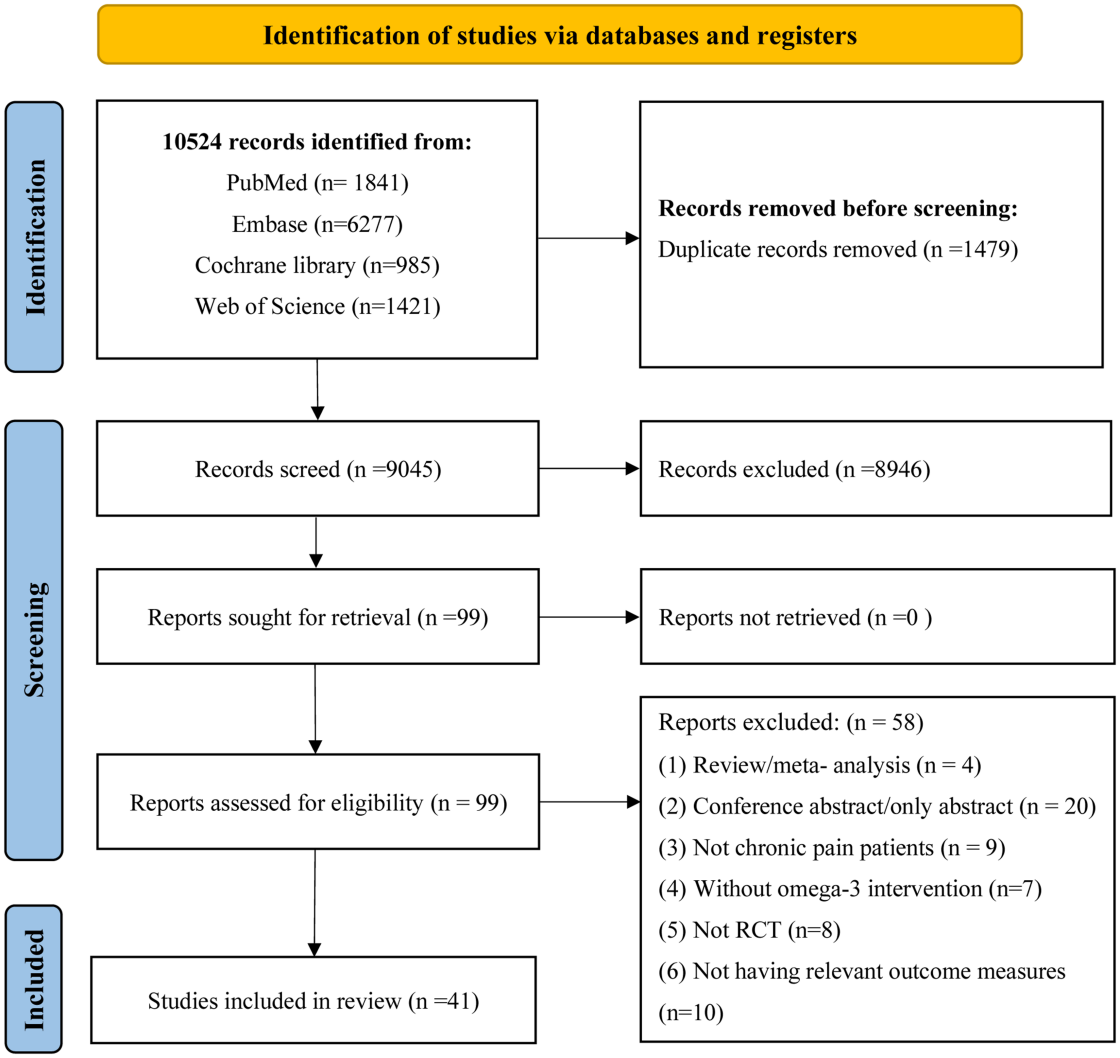
Flow chart of article retrieval.

The selection process, along with excluded records and reasons for exclusion, is outlined in Figure 1. The key characteristics of the included studies are presented in Table 1. Among the 99 full-text articles evaluated, 58 were excluded for various reasons, including 4 that were reviews or meta-analyses, 20 that were conference abstracts, 9 that did not involve patients with chronic pain, 7 that did not incorporate n-3 fatty acid intervention, 8 with non-RCT, and 10 that lacked relevant outcome measures. Therefore, 41 randomized controlled trials were retained for the final analysis (18–58).

**Table 1:** Characteristics of study included in meta-analysis.

Study	Country	Study design	Population (treatment/control)	Mean age (years) (intervention/control)	Intervention group	Control group	Disease	Duration	Daily dose	Outcome score
Möller et al. (18)	Spain	RCT	23/28	61.2/57.3	SPMs (derive from essential PUFAs, namely AA, EPA, and DHA	Olive oil	Symptomatic knee osteoarthritis	12 weeks	500 mg × 4/day (weeks 1–6), then 500 mg × 2/day (weeks 7–12)	VAS
Faurot et al. (19)	USA	RCT	47/47	38.8/36.9	A high n-3, an average n-6 diet	Average intakes of n-3 and n-6 fatty acids	Migraine	16 weeks	NA	PROMIS-29
			46/47	39.4/36.9	A high n-3, low n-6 diet				NA	
Pérez-Piñero et al. (20)	Spain	RCT	31/30	51.1/50.2	AvailOm® 50 High EPA	Placebo	Persistent knee pain	8 weeks	EPA/DHA-lysine salts (25%)	WOMAC Score
Carlisle et al. (21)	USA	RCT	12/13	55.2/ 55.1	Calamari oil (n-3 fatty acids)	Placebo	Self-reported mixed pain (e.g., bone/muscle + back, joint + back, or cervical + joint pain)	12 weeks	N-3 fatty acids: 230 mg (DHA 130 mg, EPA 55 mg)	NPRS-11
Sasahara et al. (22)	Japan	RCT	60/60	40.3/41.4	L-Serine and EPA	Placebo	Chronic low-back and knee pain	8 weeks	594 mg L-Ser and 149 mg EPA	BPI
MacFarlane et al. (23)	Spain	RCT	595/626	67.9/ 67.6	N-3 fatty acids (Omacor®)	Placebo	Knee pain	Mean: 5.3 years	Omacor® 1 g/d (EPA + DHA 840 mg, 1.3:1 ratio)	WOMAC Score
Nodler et al. (24)	USA	RCT	17/19	18.9/ 20.1	Fish oil	Placebo	Endometriosis	6 months	Fish oil 1,000 mg/d [ω-3 FAs 720 mg: EPA 488 mg, DHA 178 mg]	VAS
Godazandeh et al. (25)	Iran	RCT	51/49	NA	Flaxseed oil 1,000 mg/d (soft capsule)	Vitamin E	Fibrocystic breast: mastalgia + nodularity	2 months	α-Linolenic acid 350 mg/d	VAS
Hadian et al. (26)	USA	RCT	20/20	37.7/37.8	N-3 fatty acids	Placebo	RAS	6 months	180 mg of EPA and 120 mg of DHA	VAS
Stonehouse et al. (27)	Australia	RCT	117/118	55.8/ 56.0	Krill oil	Mixed vegetable oil	Osteoarthritic knee pain	6 months	600 mg of EPA, 280 mg of DHA, and 0.45 mg of astaxanthin	WOMAC Score
Kuszewski et al. (28)	Australia	RCT	32/31	65.4/65.4	Fish oil	Placebo	Osteoarthritis	16 weeks	2,000 mg of DHA and 400 mg of EPA per day	VAS
Lustberg et al. (29)	USA	RCT	22/22	59.5/ 57.8	N-3 fatty acids	Placebo	Musculoskeletal pain	24 weeks	2,580 mg EPA and 1,380 mg DHA	5-point score
Noguchi et al. (30)	Japan	RCT	45/54	39.2/40.6	N-3 fatty acids	Placebo	Traumatic injury	12 weeks	1,470 mg DHA and 147 mg EPA	SF-36
Hill et al. (31)	Australia	RCT	85/83	61.0/61.0	Blend of fish oil and sunola oil	High-dose fish oil	Knee osteoarthritis	24 months	4.5 g omega-3 fatty acids or 0.45 g omega-3 fatty acids	WOMAC Score
Ramsden et al. (32)	USA	RCT	32/32	41.0/42.0	Low n-6: high n-3 ratio	Low n-6	Headache pain	12 weeks	Increased EPA and DHA intake	HIT-6
Blommers et al. (33)	Netherlands	RCT	30/30	39.6/36.8	Fish oil	Corn oil + wheat-germ oil	Chronic mastalgia	6 months	1,128 mg EPA, 714 mg DHA	4-point score
Ramsden et al. (34)	USA	RCT	33/34	NA	Low n-6: high n-3 ratio	Low n-6	Headache pain	12 weeks	NA	HIT-6
El Khouli et al. (35)	Egypt	RCT	25/25	33.7/32.5	N-3 fatty acids	Placebo	RAS	6 months	Capsules of 1,000 mg each/day	VAS
Das Gupta et al. (36)	Bangladesh	RCT	40/41	49.9/44.7	N-3 fatty acids	indomethacin	Rheumatoid arthritis	12 weeks	Indomethacin (75 mg) along with omega-3 fatty acids (3 g)	VAS
Park et al. (37)	Korea	RCT	41/40	49.2/47.6	N-3 fatty acids	Placebo	Rheumatoid arthritis	16 weeks	2,090 mg of EPA and 1,165 mg of DHA per day	Pain scale
Rahbar et al. (38)	Iran	RCT	47/48	20.0/19.8	N-3 fatty acids	Placebo	Primary dysmenorrhea	3 months	180 mg of EPA and 120 mg of DHA per day	VAS
Caturla et al. (39)	Spain	RCT	23/22	39.2/39.9	Fish oil and standardized lemon verbena extract	Placebo	Joint discomfort/pain	9 weeks	1233.6 mg EPA + 986.4 mg DHA/day (Weeks 1–5); 616.8 mg EPA + 493.2 mg DHA/day (Weeks 6–9)	WOMAC Score
Galarraga et al. (40)	UK	RCT	49/48	58.0/61.0	Cod liver oil (n-3 fatty acids)	Placebo	Rheumatoid arthritis	9 months	1,500 mg EPA + 700 mg DHA/day	VAS
Harel et al. (41)	USA	RCT	14/13	NA	N-3 fatty acids	Placebo	Recurrent migraines	2 months	756 mg of EPA and 498 mg of DHA per day	Seven-point faces pain scale
Brunborg et al. (42)	Norway	RCT	18/20	48.1/47.7	Cod liver oil	Seal oil	Inflammatory bowel disease and joint pain	14 days	2.3 g EPA, 0.3 g DPA, and 3.7 g DHA per day	VAS
Bjørkkjaer et al. (43)	Norway	RCT	10/9	NA	Seal oil	Soy oil	Inflammatory bowel disease-related joint pain	10 days	2.0 g EPA, 0.9 g DPA and 2.2 g DHA per day	VAS
Hansen et al. (44)	Denmark	RCT	36/45	59.0/54.0	Fish meal	Normal diet	Rheumatoid arthritis	6 months	600 mg EPA, 420 mg DHA per day	VAS
Nordström et al. (45)	Finland	RCT	11/11	51.0/53.0	Alpha-LNA	Placebo	Rheumatoid arthritis	3 months	30 g of flaxseed oil (32% alpha-LNA)	VAS
Berbert et al. (46)	Brazil	RCT	17/13	51.0/48.0	Fish oil n-3 fatty acids + olive oil	Soy oil	Rheumatoid arthritis	24 weeks	3 g/d fish oil n-3 fatty acids,6.8 g oleic acid	5-point score
			13/13	51.0/48.0	Fish oil n-3 fatty acids				3 g/d fish oil n-3 fatty acids	
Remans et al. (47)	Netherlands	RCT	26/29	59.5/52.9	PUFA supplement drink	Placebo drink	Rheumatoid arthritis	4 months	1,400 mg EPA, 200 mg DHA- 500 mg GLA	VAS
Kawabata et al. (48)	Japan	RCT	11/9	23.3/27.1	Fish oil	Middle chain triglycerides (edible oil)	Asthenopia (eye-pain, low back pain, headache)	4 weeks	162 mg EPA, 783 mg DHA	VAS
Stammers et al. (49)	United Kingdom	RCT	29/29	67.0/69.0	Current NSAIDs + cod liver oil	Current NSAIDs + olive oil	Osteoarthritis	6 months	786 mg EPA	VAS
Geusens et al. (50)	Belgium	RCT	19/20	56.0/59.0	Fish oil	Olive oil	Rheumatoid arthritis	12 months	1,680 mg EPA, 360 mg DHA	5-point score
Nielsen et al. (51)	Denmark	RCT	27/24	NA	Fish oil	Average diet	Rheumatoid arthritis	12 weeks	2000 mg EPA, 1200 mg DHA	VAS
Kremer et al. (52)	USA	RCT	20/12	59.0/58.0	Fish oil	Olive oil	Rheumatoid arthritis	24 weeks	27 and 18 mg/kg/day of EPA and DHA	5-point score
			17/12	58.0/58.0					54 and 36 mg/kg/day of EPA and DHA	
Van der Tempel et al. (53)	Netherlands	RCT	8/8	NA	Fish oil	Coconut oil	Rheumatoid arthritis	12 weeks	12 capsules of fractionated fish oil	4-point score
Sundrarjun et al. (54)	Thailand	RCT	23/23	46.2 /46.0	Low n-6 diet + fish oil	Low n-6 diet + placebo	Rheumatoid arthritis	12 weeks	1880 mg EPA, 1480 mg DHA	VAS
Kremer et al. (55)	USA	RCT	15/14	58.0/57.0	Diclofenac+ fish oil + corn oil	Diclofenac + corn oil	Rheumatoid arthritis	48 weeks	130 mg/kg/d of n-3 (44% EPA - 24%DHA)	5-point score
Skoldstam et al. (56)	Sweden	RCT	22/21	58.0/55.0	Fish oil	Inactive oil (maize, olive and peppermint oils)	Rheumatoid arthritis	6 months	1800 mg EPA, 1200 mg DHA	4-point score
Magarò et al. (57)	Italy	RCT	10/10	NA	Diclofenac + n-3 fatty acids	Diclofenac	Rheumatoid arthritis	45 days	1,600 mg EPA, 1100 mg DHA	VAS
Adam et al. (58)	Germany	RCT	30/30	58.0/56.8	Fish oil	Placebo	Rheumatoid arthritis	3 months	30 mg/kg/day of total n-3 fatty acids	VAS

RCT, randomized controlled trial; n-3 fatty acids, omega-3 fatty acids; PUFA, polyunsaturated fatty acid; NSAIDs, non-steroidal anti-inflammatory drugs; SPMs, specialized pro-resolving mediators; AA, arachidonic acid; EPA, eicosapentaenoic acid; DHA, docosahexaenoic acid; DPA, docosapentaenoic acid; PROMIS-29, Patient-Reported Outcomes Measurement Information System; WOMAC, Western Ontario and McMaster Universities Osteoarthritis Index; NPRS-11, 11-Point Numeric Pain Rating Scale; BPI, brief pain inventory; VAS, Visual Analogue Scale; SF-36, 36-Item Short Form Health Survey; HIT-6, Headache Impact Test; RAS, recurrent aphthous stomatitis; LNA, linolenic acid; GLA, gamma-linolenic acid.

Among the 41 RCTs, 26 trials (18–20, 22–25, 27–32, 34, 35, 40, 41, 43, 45–48, 51, 53, 54, 58) (63.4%) were classified as low risk of bias, 11 trials (21, 26, 37–39, 42, 44, 50, 52, 56, 57) (26.8%) had some concerns, and four trials (33, 36, 49, 55) (9.8%) were classified as high risk of bias. Refer to the Supplementary material for detailed assessments.

In the domain of bias arising from the randomization process, 37 trials (18–20, 22–25, 27–35, 37–41, 43–58) (90.2%) were classified as low risk, as they clearly described adequate random sequence generation and allocation concealment using computer-based or equivalent methods. The remaining four trials (21, 26, 36, 42) (9.8%) lacked sufficient information to confirm proper randomization procedures and were thus classified as having “some concerns.” Regarding bias due to deviations from intended interventions, the majority of studies complied with the protocol and maintained appropriate blinding. All 41 trials (18–58) (100%) were classified as low risk in this domain. In the domain of bias due to missing outcome data, 33 trials (18–32, 34, 35, 37, 38, 40–43, 45–48, 51, 53, 54, 56–58) (80.5%) had a loss-to-follow-up rate below 5% and used appropriate methods for handling missing data, and were classified as low risk. Four trials (39, 44, 50, 52) (9.8%) had >5% missing data but employed valid imputation strategies or provided transparent explanations, leading to “some concerns.” Another four trials (33, 36, 49, 55) (9.8%) had substantial missing data without adequate justification or handling, and were thus classified as high risk. For bias in the measurement of outcomes, all 41 trials (18–58) (100%) were classified as low risk, as outcome assessments were performed by blinded assessors or used objective validated instruments. In the domain of bias in the selection of the reported result, 32 trials (18–35, 39–43, 45–48, 50, 51, 53, 54, 58) (78.0%) reported prespecified outcomes consistent with protocols or registries, while nine trials (36–38, 44, 49, 52, 55–57) (22.0%) were classified as having “some concerns” due to unclear analytical plans or selective reporting.

### 3.2 Primary outcome: effect of omega-3 fatty acids on pain intensity

**Figure 2:**
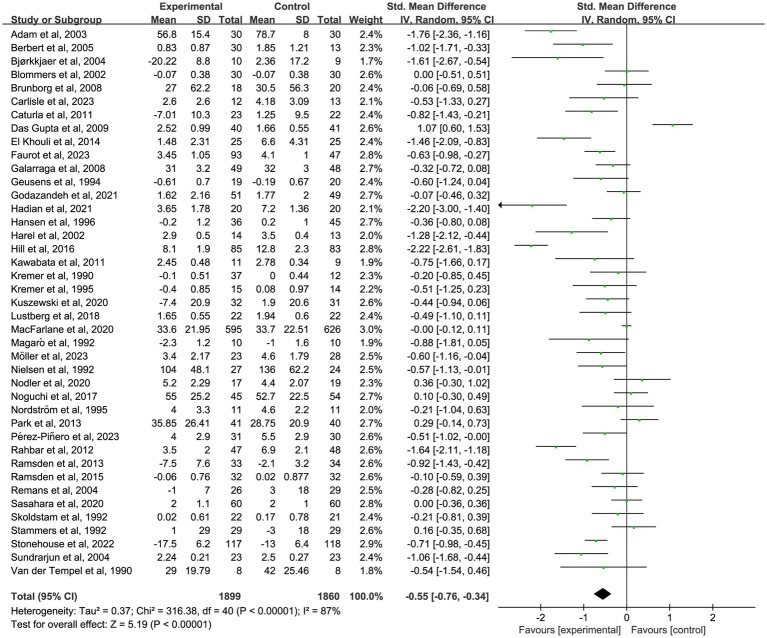
Forest plot of the effect of omega-3 fatty acid supplementation on chronic pain. Random-effects model was used to compute pooled SMDs with 95% CI based on 41 studies (*n* = 3,759).

The primary analysis sought to evaluate the impact of omega-3 fatty acid supplementation on pain intensity compared to control conditions. A total of 41 randomized controlled trials (18–58) with 3,759 participants demonstrated a significant reduction in chronic pain associated with omega-3 fatty acids (SMD = −0.55; 95% CI: −0.76 to −0.34; *p* < 0.001; *I*^2^ = 87%). See Figure 2 and Table 2.

**Table 2:** Meta-analysis of intervention studies in chronic pain.

Variables	N of studies	SMD Fixed effects (95% CI)	SMD random effects (95% CI)	Heterogeneity *I*^2^%	*Q*-test *p*-value
Main analysis	41	−0.34 (−0.40, −0.27)	−0.55 (−0.76, −0.34)	87	<0.001
1-month analysis	9	−0.24 (−0.39, −0.08)	−0.27 (−0.48, −0.05)	42	0.01
2-month analysis	10	−0.36 (−0.53, −0.19)	−0.39 (−0.61, −0.18)	37	<0001
3-month analysis	22	−0.45 (−0.56, −0.35)	−0.51 (−0.87, −0.15)	91	0.005
6-month analysis	14	−0.83 (−0.96, −0.70)	−0.83 (−1.22, −0.45)	87	<0.001
Subgroup					
Disease					
Rheumatoid arthritis	16	−0.32 (−0.46, −0.17)	−0.42 (−0.76, −0.09)	80	0.01
Osteoarthritis	5	−0.88 (−1.06, −0.70)	−0.77 (−1.55, 0.00)	94	0.05
Migraine	2	−0.73 (−1.06, −0.40)	−0.84 (−1.44, −0.24)	49	0.006
Mastalgia	2	−0.04 (−0.35, 0.27)	−0.04 (−0.35, 0.27)	0	0.78
Others diseases	16	−0.21 (−0.29, −0.12)	−0.61 (−0.94, −0.29)	87	<0.001
Fatty acid type					
Fish oil	15	−0.78 (−0.93, −0.64)	−0.69 (−1.09, −0.29)	86	<0.001
N-3 fatty acids	15	−0.18 (−0.27, −0.09)	−0.55 (−0.91, −0.19)	90	0.002
Other mixed supplements	11	−0.36 (−0.50, −0.22)	−0.34 (−0.58, −0.09)	59	0.006
Country					
USA	10	−0.57 (−0.75, −0.39)	−0.62 (−0.98, −0.25)	73	0.001
Other countries	31	−0.30 (−0.37, −0.23)	−0.53 (−0.77, −0.28)	89	<0.001
Intervention period (overall)					
≥3 months	32	−0.34 (−0.41, −0.26)	−0.54 (−0.78, −0.29)	90	<0.001
<3 months	9	−0.35 (−0.54, −0.16)	−0.53 (−0.87, −0.20)	62	0.002
Daily dose					
≤1.35 g	12	−0.16 (−0.26, −0.07)	−0.60 (−0.99, −0.21)	89	0.003
>1.35 g	29	−0.52 (−0.62, −0.43)	−0.53 (−0.78, −0.28)	85	<0.001
Outcome score					
VAS score	20	−0.48 (−0.60, −0.35)	−0.60 (−0.95, −0.26)	86	<0.001
WOMAC score	5	−0.28 (−0.37, −0.18)	−0.85 (−1.63, −0.07)	97	0.03
Composite score	16	−0.29 (−0.43, −0.16)	−0.36 (−0.57, −0.14)	59	0.001
Control type					
Non-placebo control	20	−0.52 (−0.64, −0.41)	−0.50 (−0.84, −0.16)	88	0.004
Placebo control	21	−0.24 (−0.32, −0.16)	−0.59 (−0.86, −0.32)	86	<0.001

SMD, standardized mean difference; CI, confidence interval; VAS, Visual Analogue Scale; USA, United States of America; WOMAC, Western Ontario and McMaster Universities Arthritis Index. *p* < 0.05 was considered statistically significant. Some studies contributed data to more than one duration category (1, 2, 3, or 6 months). Therefore, the groups are not mutually exclusive, and the total count exceeds the number of unique studies.

**Figure 3:**
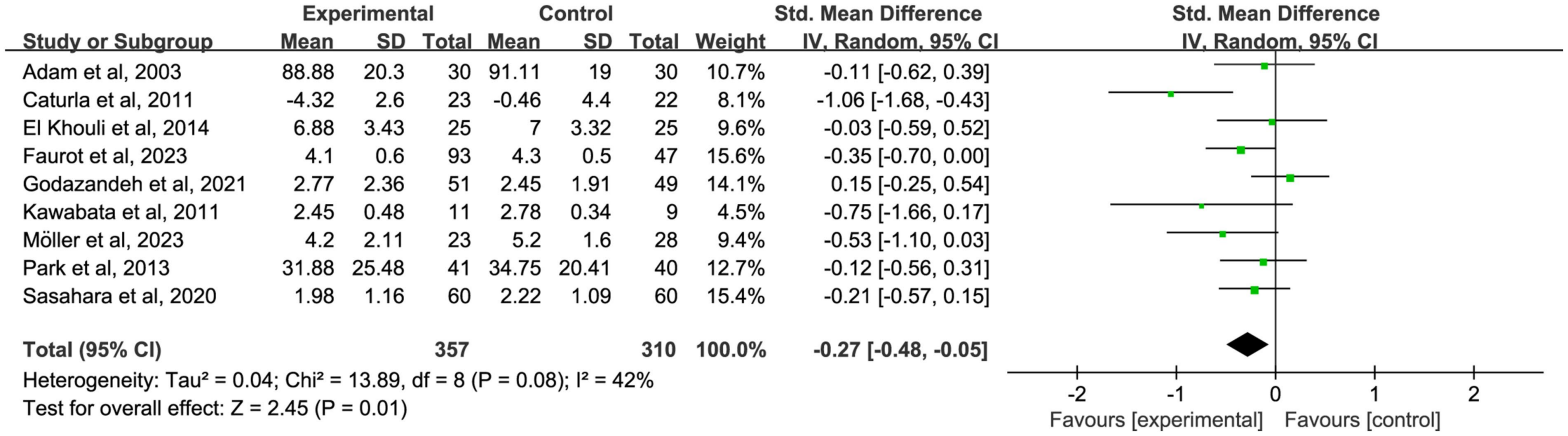
Forest plot of the effect of omega-3 fatty acid supplementation on chronic pain at 1 month. A random-effects model was used to compute pooled SMDs with 95% CI based on nine studies (*n* = 667).

**Figure 4:**
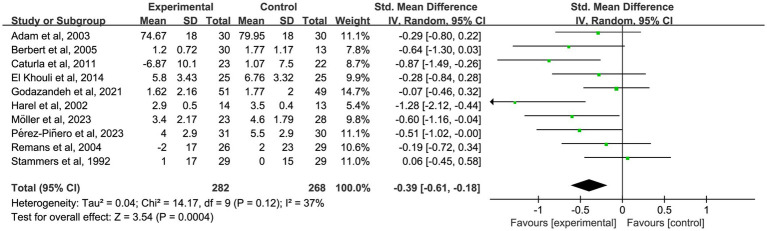
Forest plot of the effect of omega-3 fatty acid supplementation on chronic pain at 2 months. A random-effects model was used to compute pooled SMDs with 95% CI based on 10 studies (*n* = 550).

**Figure 5:**
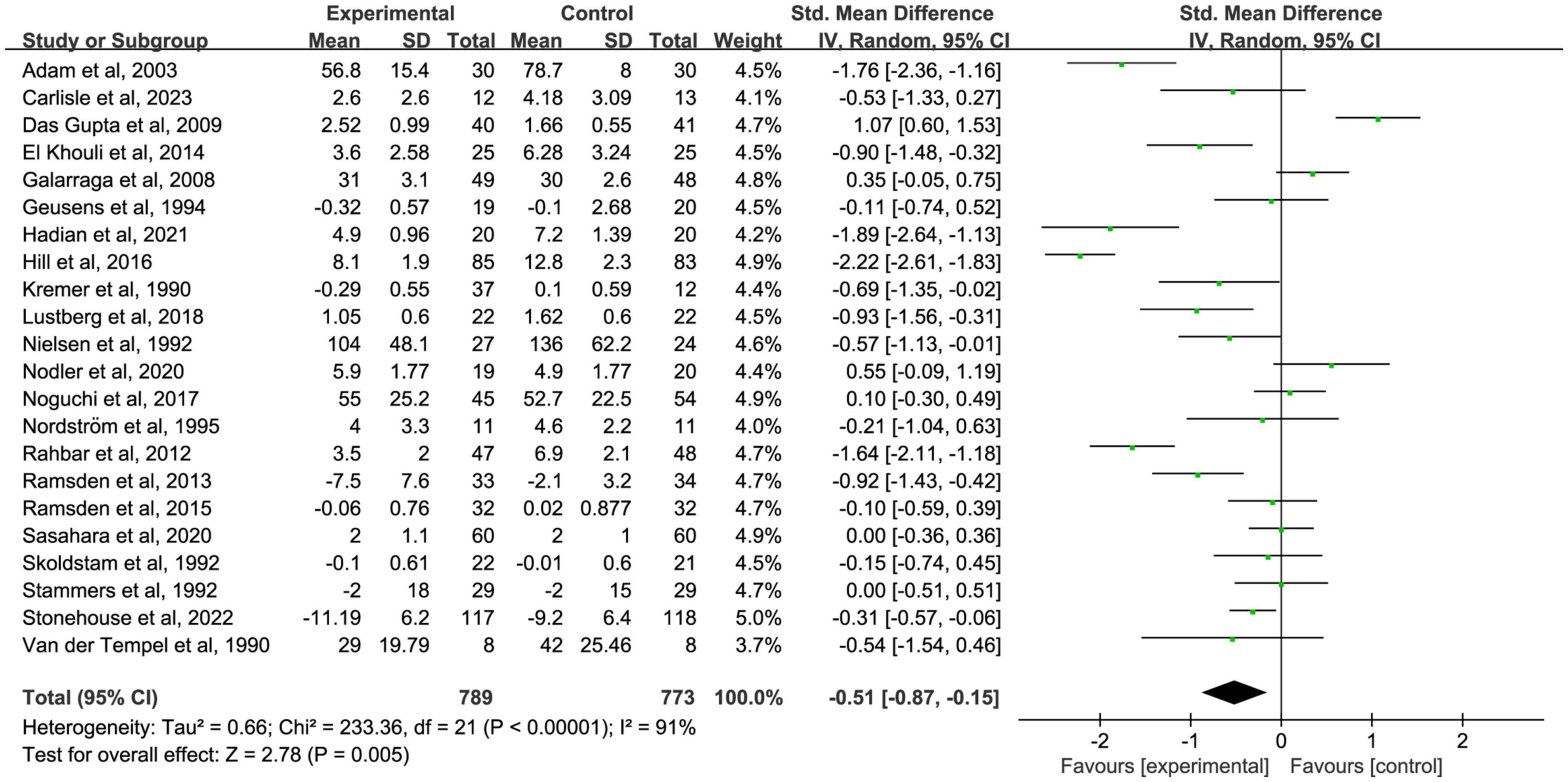
Forest plot of the effect of omega-3 fatty acid supplementation on chronic pain at 3 months. A random-effects model was used to compute pooled SMDs with 95% CI based on 22 studies (*n* = 1,562).

**Figure 6:**
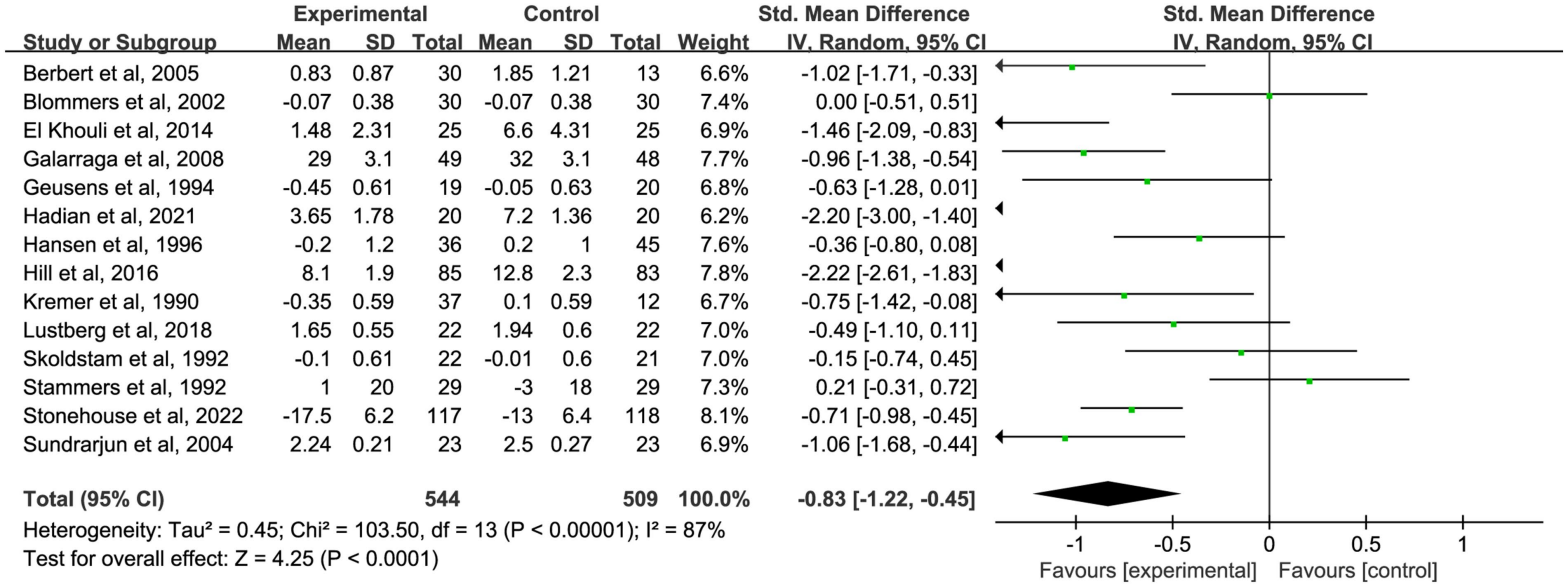
Forest plot of the effect of omega-3 fatty acid supplementation on chronic pain at 6 months. A random-effects model was used to compute pooled SMDs with 95% CI based on 14 studies (*n* = 1,053).

To further investigate the time-dependent effects of omega-3 fatty acid supplementation, an exploratory subgroup analysis was conducted, stratified by intervention duration (1, 2, 3, and 6 months). Certain trials reported pain outcomes at multiple time points, indicating that some studies contributed data to more than one subgroup. These results are intended to illustrate potential trends over time, not to be used for direct comparisons between durations. In nine studies (18, 19, 22, 25, 35, 37, 39, 48, 58) with 1-month outcomes, omega-3 fatty acid supplementation led to a significant reduction in chronic pain (*n* = 667, SMD = −0.27, 95% CI: −0.48 to −0.05, *p* = 0.01; *I*^2^ = 42%). See Figure 3 and Table 2. In 10 studies (18, 20, 25, 35, 39, 41, 46, 47, 49, 58) that reported 2-month outcomes, a similar impact was noted (*n* = 550, SMD = −0.39, 95% CI: −0.61 to −0.18, *p* < 0.001; *I*^2^ = 37%). See Figure 4 and Table 2. In 22 studies (21, 22, 24, 26, 27, 29–32, 34–36, 38, 40, 44, 45, 49–53, 56) with 3-month outcomes, pain scores were also significantly improved (*n* = 1,562, SMD = −0.51, 95% CI: −0.87 to −0.15, *p* = 0.005; *I*^2^ = 91%). See Figure 5 and Table 2. In 14 studies (26, 27, 29, 31, 33, 35, 40, 44, 46, 49, 50, 52, 54, 56) reporting 6-month outcomes, the analgesic effect became more pronounced (*n* = 1,053, SMD = −0.83, 95% CI: −1.22 to −0.45, *p* < 0.001; *I*^2^ = 87%). See Figure 6 and Table 2. The meta-regression results demonstrated a significant positive relationship between follow-up duration and analgesic efficacy. Specifically, for each additional month of intervention, the analgesic effect size increased by a factor of 10.3% [exp(b) = 1.103, 95% CI = (1.008, 1.207), *p* = 0.033]. This finding indicates that as the duration of omega-3 fatty acid intervention increases, analgesic efficacy improves significantly.

### 3.3 Subgroup analyses

**Figure 7:**
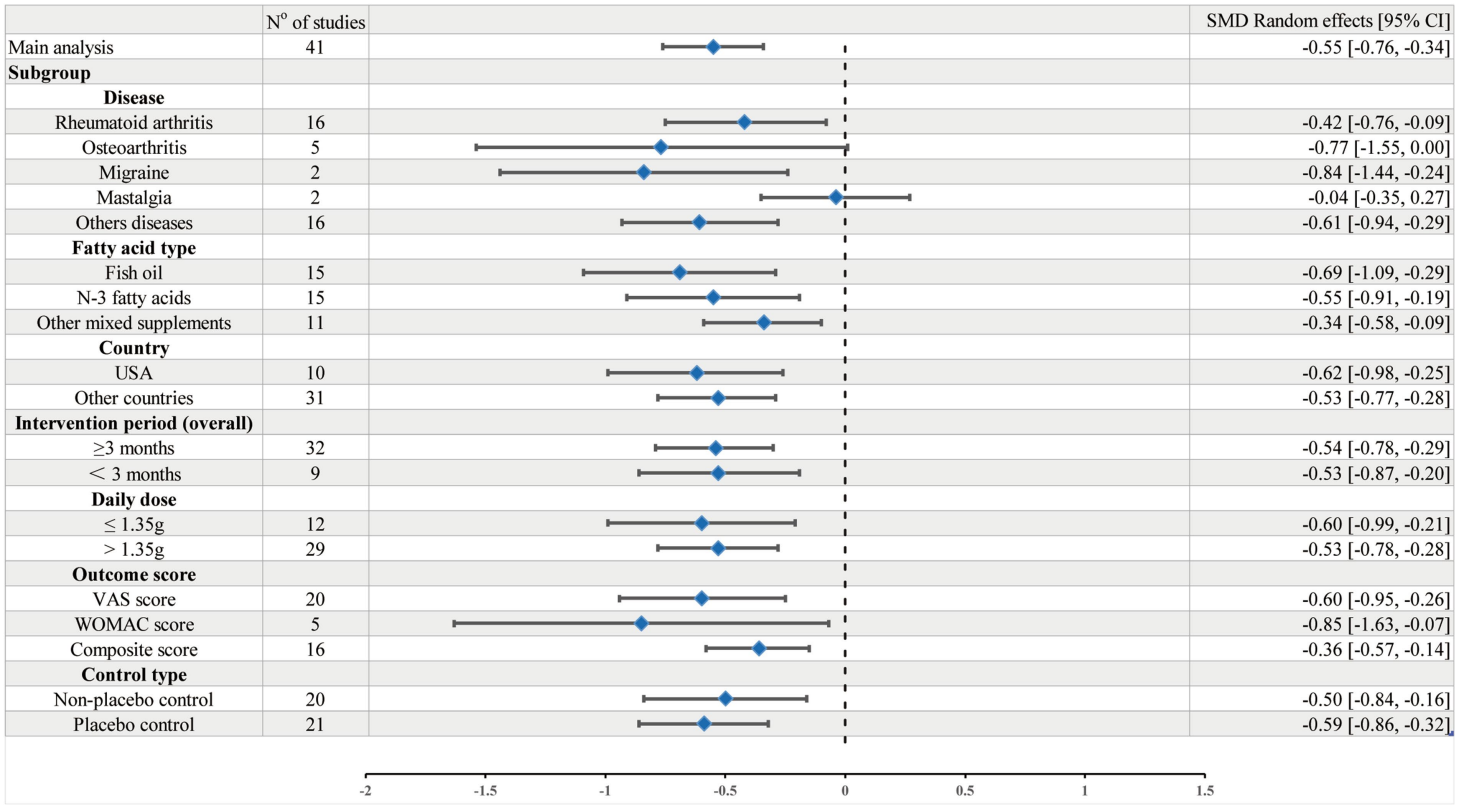
Forest plot of the pooled and subgroup effects of omega-3 fatty-acid supplementation on chronic-pain intensity (random-effects model).

Forest plot depicting the pooled and subgroup effects of omega-3 fatty acid supplementation on chronic pain intensity (random-effects model). See Figure 7.

#### 3.3.1 Pain assessment tools

In 20 studies (18, 24–26, 28, 35, 36, 38, 40, 42–45, 47–49, 51, 54, 57, 58) utilizing the VAS, omega-3 fatty acid supplementation led to a significant reduction in pain intensity (*n* = 1,083; SMD = −0.60; 95% CI: −0.95 to −0.26; *p* < 0.001; *I*^2^ = 86%). A similar analgesic effect was observed in 16 studies (19, 21, 22, 29, 30, 32–34, 37, 41, 46, 50, 52, 53, 55, 56) employing other validated instruments (*n* = 946; SMD = −0.36; 95% CI: −0.57 to −0.14; *p* = 0.001; *I*^2^ = 59%). Importantly, of these, five studies (20, 23, 27, 31, 39) utilized the WOMAC, which also demonstrated a statistically significant effect (*n* = 1730; SMD = −0.85; 95% CI: −1.63 to −0.07; *p* = 0.03; *I*^2^ = 97%), despite high heterogeneity, as detailed in Supplementary material and Table 2.

#### 3.3.2 Disease type

Omega-3 fatty acid supplementation significantly alleviated pain in patients with rheumatoid arthritis (RA), based on data from 16 studies (36, 37, 40, 44–47, 50–58) (*n* = 813, SMD = −0.42, 95% CI: −0.76 to −0.09, *p* = 0.01, *I*^2^ = 80%). A significant benefit was also observed in migraine patients, derived from pooled data from two studies (19, 41) (*n* = 167, SMD = −0.84, 95% CI: −1.44 to −0.24, *p* = 0.006, *I*^2^ = 49%). Similarly, a moderate effect size was observed in the category of “other chronic pain conditions,” derived from 16 studies (20–24, 26, 29, 30, 32, 34, 35, 38, 39, 42, 43, 48) (*n* = 2044, SMD = −0.61, 95% CI: −0.94 to −0.29, *p* < 0.001, *I*^2^ = 87%). Neither the osteoarthritis (OA) nor mastalgia subgroup showed a statistically significant benefit in the random-effects model. In OA, no significant analgesic effect was observed, derived from 5 trials (18, 27, 28, 31, 49) (*n* = 575, SMD = −0.77, 95% CI: −1.55 to 0.00, *p* = 0.05, *I*^2^ = 94%). Likewise, mastalgia showed no analgesic advantage, derived from 2 trials (25, 33) (*n* = 160, SMD = −0.04, 95% CI: −0.35 to 0.27, *p* = 0.78, *I*^2^ = 0%), as detailed in Supplementary material and Table 2.

#### 3.3.3 Intervention duration

Subgroup analysis based on intervention duration revealed that both short-term (<3 months) and long-term (≥3 months) supplementation with omega-3 fatty acids led to significant reductions in pain. Short-term interventions (nine studies) (20, 22, 39–43, 48, 57) demonstrated a statistically significant effect (*n* = 450, SMD = −0.53, 95% CI: −0.87 to −0.20, *p* = 0.002; *I*^2^ = 62%), albeit smaller than long-term interventions (32 studies) (18, 19, 21, 23–38, 44–47, 49–56, 58) which showed a stronger analgesic effect (*n* = 3,309, SMD = −0.54, 95% CI: −0.78 to −0.29, *p* < 0.001; *I*^2^ = 90%). as shown in Supplementary material and Table 2.

#### 3.3.4 Country

Omega-3 fatty acid supplementation was found to significantly reduce pain in both studies conducted in the United States (10 studies (19, 21, 24, 26, 29, 32, 34, 41, 52, 55); *n* = 521, SMD = −0.62, 95% CI: −0.98 to −0.25, *p* = 0.001; *I*^2^ = 73%) and those from other countries (31 studies (18, 20, 22, 23, 25, 27, 28, 30, 31, 33, 35–40, 42–51, 53, 54, 56–58); *n* = 3,238, SMD = −0.53, 95% CI: −0.77 to −0.28, *p* < 0.001; *I*^2^ = 89%). Despite moderate to high heterogeneity, the consistent effect sizes across regions suggest that omega-3’s pain-relieving benefits are applicable to diverse populations and healthcare systems as shown in Supplementary material and Table 2.

#### 3.3.5 Type of unsaturated fatty acid supplementation

Subgroup analysis based on the type of unsaturated fatty acid supplementation showed varying analgesic efficacy. Among 15 studies (24, 28, 31, 33, 39, 44, 46, 48, 50–54, 56, 58) on fish oil, a significant reduction in pain was observed (*n* = 820; SMD = −0.69; 95% CI: −1.09 to −0.29; *p* < 0.001; *I*^2^ = 86%). Similarly, 15 studies (19, 21, 23, 26, 29, 30, 32, 34–38, 40, 41, 57) on omega-3 fatty acids also demonstrated a significant analgesic effect (*n* = 2,151; SMD = −0.55; 95% CI: −0.91 to −0.19; *p* = 0.002; *I*^2^ = 90%). Additionally, 11 studies (18, 20, 22, 25, 27, 42, 43, 45, 47, 49, 55) on mixed supplement formulations (including combined n-3 and other fatty acids) reported a comparable reduction in pain (*n* = 788; SMD = −0.34; 95% CI: −0.58 to −0.09; *p* = 0.006; *I*^2^ = 59%). as shown in Supplementary material and Table 2.

#### 3.3.6 Omega-3 dosage

Both dosage groups showed significant reductions in pain intensity compared to control. However, the low-dose group (≤1.35 g/day) exhibited greater analgesic effects in 12 trials (21–26, 35, 38, 41, 44, 48, 49) (*n* = 1,873; SMD = −0.60; 95% CI: −0.99 to −0.21; *p* = 0.003; *I*^2^ = 89%), while the high-dose group (>1.35 g/day) showed a more modest effect in 29 trials (18–20, 27–34, 36, 37, 39, 40, 42, 43, 45–47, 50–58) (*n* = 1,886; SMD = −0.53; 95% CI: −0.78 to −0.28; *p* < 0.001; *I*^2^ = 85%). as shown in Supplementary material and Table 2.

#### 3.3.7 Placebo-controlled vs. active-controlled trials

Omega-3 fatty acid supplementation showed a significant analgesic effect compared to placebo in 21 randomized trials (20–24, 26, 28–30, 35, 37–41, 44, 45, 47, 51, 54, 58) (*n* = 2,419; SMD = −0.59; 95% CI: −0.86 to −0.32; *p* < 0.001; *I*^2^ = 86%). Out of the 20 trials (18, 19, 25, 27, 31–34, 36, 42, 43, 46, 48–50, 52, 53, 55–57) with an active comparator, a statistically significant effect was also observed (*n* = 1,340; SMD = −0.50; 95% CI: −0.84 to −0.16; *p* = 0.004; *I*^2^ = 88%). as shown in Supplementary material and Table 2.

### 3.4 Sensitivity analysis and publication bias

The distribution of study weights was assessed, and no single study significantly impacted the overall pooled effect. Each study contributed relatively equally, with a weight range of 1%–5%, and no outliers were identified. Sensitivity analysis was performed by omitting one study at a time, and the results remained stable, confirming the robustness of the findings.

Publication bias was assessed using a funnel plot, as shown in Supplementary material, which exhibited slight asymmetry with a leftward skew in the distribution of effect sizes. Egger’s test provided marginal evidence of publication bias (*p* = 0.052), whereas Begg’s test indicated statistical significance (*p* = 0.009). A trim-and-fill analysis was conducted using a linear estimator under a random-effects model to further explore this possibility. Six potentially missing studies were imputed on the right side of the funnel plot, based on the trim-and-fill method. After adjustment, the pooled effect size remained statistically significant (adjusted SMD = −0.723), suggesting that the analgesic benefit of omega-3 fatty acids is robust and minimally influenced by small-study effects or selective reporting.

## 4 Discussion

### 4.1 Principal findings

In this comprehensive meta-analysis of 41 randomized controlled trials (*N* = 3,759), Omega-3 fatty acid supplementation demonstrated a moderate, statistically and clinically significant reduction in chronic pain intensity (random-effects SMD = −0.55). Beneficial effects emerged as early as 1 month (SMD = −0.27) and were maintained at 2 months (SMD = −0.39) and 3 months (SMD = −0.51), with the largest effect at 6 months (SMD = −0.83), indicating a time-dependent, cumulative analgesic response. A clear dose pattern was evident: low-dose regimens (≤1.35 g day^−1^) yielded a better effect (SMD = −0.60) than higher doses (>1.35 g day^−1^; SMD = −0.53). Subgroup analyses confirmed robust benefit in RA, migraine and miscellaneous chronic pain conditions, while no significant improvement was detected in OA or mastalgia. Risk-of-bias assessment indicated that 63% of trials were low risk and sensitivity analyses showed that removing any single study did not dramatically alter the pooled estimate; trim-and-fill procedures suggested minimal impact of publication bias.

### 4.2 Comparison with previous work

Recent systematic reviews and meta-analyses have come together to support the therapeutic benefits of omega-3 fatty acids supplementation for specific chronic pain conditions. Goldberg et al. (59), pooling 17 randomized controlled trials, demonstrated that omega-3 fatty acids significantly attenuate patient-reported pain intensity in inflammatory disorders such as RA. Concordantly, a 2025 systematic review reported a robust analgesic effect of omega-3 fatty acids in migraine, reflected by a significant reduction in standardized headache-severity scores (60). These observations closely align with the subgroup findings in this study—RA and migraine—thereby reinforcing the reliability and reproducibility of our results across studies. Mechanistically, the analgesic actions of omega-3 fatty acids stem from their multi-tiered modulation of the inflammatory cascade. First, incorporation of omega-3 fatty acids into membrane phospholipids displaces arachidonic acid, lowering biosynthesis of key pronociceptive eicosanoids such as prostaglandin E₂ and leukotriene B₄ (61). Second, omega-3 fatty acids are enzymatically converted to specialized pro-resolving mediators—for example, resolvins, protectins, and maresins—which engage receptors such as FPR2/ALX and ChemR23 to suppress NF-κB signaling and down-regulate pro-inflammatory cytokines (TNF-α, IL-1β, IL-6) (62). Third, omega-3 fatty acids promote macrophage polarization toward the anti-inflammatory M2 phenotype, thereby accelerating active resolution of inflammation (63). Collectively, these anti-inflammatory and neuro-modulatory mechanisms intercept the pathological continuum from peripheral tissue inflammation to central sensitization (64), providing a compelling molecular rationale for integrating omega-3 fatty acids into contemporary chronic-pain management paradigms.

In the present subgroup analyses, omega-3 fatty acids supplementation did not demonstrate a statistically significant analgesic effect in patients with OA or mastalgia. Several factors may account for this null finding. First, OA is characterized by a relatively low-grade inflammatory profile compared to conditions such as RA (65), thereby potentially limiting the therapeutic scope for omega-3’s anti-inflammatory mechanisms. Second, the pathophysiology of OA-related pain is largely mechanical in origin, driven by cartilage wear, subchondral bone changes, and joint loading, with central sensitization often contributing to chronic symptom persistence (66, 67). These mechanisms may be less responsive to lipid-mediated anti-inflammatory modulation. Third, outcome assessment tools may influence effect detection. Most OA trials employed the WOMAC index, which combines pain, stiffness, and physical function subdomains. The multidimensional nature of WOMAC may dilute changes in pain-specific outcomes, especially when compared to more sensitive, unidimensional measures like the VAS. Taken together, these biological and methodological factors may explain the absence of a statistically significant analgesic effect of omega-3 in OA trials. As for mastalgia, the condition is largely hormonally mediated, primarily influenced by cyclical fluctuations in estrogen and prolactin levels rather than inflammatory pathways (68).

### 4.3 Clinical implications

In this meta-analysis, the pooled SMD was −0.55. When back-translated to a 0–100 mm VAS using a standard deviation of 20–25 mm—commonly reported in chronic pain trials—this corresponds to an absolute pain reduction of approximately 11–14 mm. At the 6-month follow-up, an SMD of −0.83 equates to a reduction of roughly 17–21 mm on the VAS. Given that the minimal clinically important difference (MCID) for chronic pain in adults is generally considered to be ~10 mm (69), the effects observed in this analysis exceed the threshold for clinical relevance.

For comparison, the established analgesic dose of oral diclofenac (150 mg/day) yields a pooled effect size of SMD –0.56, corresponding to an approximate 14-mm reduction in VAS pain scores (70). The efficacy of omega-3 fatty acid supplementation (SMD –0.55) appears broadly comparable in magnitude; however, unlike nonsteroidal anti-inflammatory drugs (NSAIDs), omega-3 s are associated with a substantially lower risk of gastrointestinal and cardiovascular toxicities. Importantly, omega-3 fatty acids should not be regarded as equivalent to NSAIDs, which remain the first-line therapy for acute pain. Rather, omega-3 s may be best positioned as a safer adjunct or as a long-term strategy in the management of chronic pain. The analgesic effect demonstrated a clear time-dependent escalation: the SMD improved from −0.27 to −0.51 at 1–3 months and reached −0.83 at 6 months. This temporal pattern aligns with the kinetics of omega-3 fatty acids merging into cell membranes, lowering the n-6: n-3 ratio, and enhancing the synthesis of SPMs such as resolvin D1 and resolvin E1. SPMs directly down-regulate nociceptive ion channels and suppress spinal glial activation, providing a “pro-resolution” form of analgesia distinct from conventional anti-inflammatory drugs (62, 71). Our subgroup analysis yielded consistent results, reinforcing the notion that longer durations of omega-3 supplementation are necessary to achieve clinically meaningful pain relief. This finding is consistent with previous reviews suggesting that prolonged supplementation is necessary to achieve clinically meaningful analgesic effects (72). A greater effect was observed in the low-dose group (≤1.35 g day^−1^; SMD = −0.60), presumably due to saturation of the plasma omega-3 fatty acids curve and better adherence relative to higher doses; nevertheless, doses >1.35 g day^−1^ remained efficacious. These findings are consistent with prior evidence suggesting that higher doses may not confer additional benefits for chronic pain relief and could even be less effective in certain contexts (31). Accordingly, dosing can be individualized on the basis of cost-effectiveness and patient tolerability.

### 4.4 Strengths

The present review surpasses earlier syntheses in several critical respects. First, by pooling 41 randomized controlled trials encompassing 3,759 participants—nearly double the sample size of the largest prior meta-analysis—and spanning migraine, RA, neuropathic pain, and musculoskeletal conditions, we markedly increased both statistical power and external validity. Second, we provide the first systematic evidence that the dose–response relationship is non-linear: daily intakes ≤1.35 g of omega-3 fatty acids produced the greatest analgesic benefit, whereas higher doses yielded diminishing returns, implying a ceiling effect or reduced adherence at large dosages. Third, we delineated the full temporal trajectory of benefit, showing that pain relief emerges within 1 month and accumulates steadily through 6 months—information that refines clinical expectations and guides future trial follow-up schedules. Fourth, the review adhered to PRISMA 2020, was prospectively registered in PROSPERO, employed the RoB 2 tool, and confirmed robustness through sensitivity, leave-one-out, and trim-and-fill analyses, thereby minimizing the risk of selective-reporting bias that troubled earlier work. Finally, comprehensive subgroup analyses revealed that that placebo-controlled trials show better effect sizes than active-control trials, underscoring the importance of comparator choice.

### 4.5 Limitations

This study has several limitations. First, substantial heterogeneity (*I*^2^ > 50%) was observed due to pooling trials with differences in pain condition, supplement formulation, dosage regimen, intervention length, and participant characteristics. Despite using random-effects models and subgroup analyses, heterogeneity remained in most comparisons, except for the first- and second-month analyses (moderate heterogeneity) and the breast-pain and migraine subgroups (relatively low heterogeneity). Second, 36.6% of trials had “some concerns” or “high” risk of bias, mainly from attrition, selective reporting, and insufficient details on randomization or allocation concealment, which may compromise internal validity. Third, potential confounders such as concurrent NSAID use and baseline omega-3 status were not consistently reported, precluding adjustment. Finally, sex-specific differences in pain response could not be examined, as most trials did not report stratified results. Future studies should better control for these factors and provide sex-disaggregated data. Nevertheless, sensitivity analyses suggested that our overall conclusions remained robust.

## 5 Conclusion

This meta-analysis demonstrates that omega-3 fatty acid supplementation produces a clinically meaningful, ceiling effect for dose escalation and time-dependent reduction in chronic pain intensity. The analgesic efficacy was most evident in inflammatory pain phenotypes such as rheumatoid arthritis and migraine, whereas evidence remains inconclusive for osteoarthritis and mastalgia. These findings support the use of omega-3 fatty acids as a safe, non-pharmacological adjunct in the management of chronic pain. Future high-quality trials are warranted to clarify the phenotype-specific indications, dose–response relationships, and long-term efficacy of omega-3 supplementation, thereby informing precision strategies for chronic pain management.

## Data Availability

The original contributions presented in the study are included in the article/Supplementary material, further inquiries can be directed to the corresponding author.
