# Supplementary material for: Effects of omega-3 fatty acids on chronic pain: a systematic review and meta-analysis
Source: Front Med (Lausanne). 2025 Nov 5;12:1654661. doi: 10.3389/fmed.2025.1654661 (PMC12627051; doi:10.3389/fmed.2025.1654661)
Supplement: Supplementary file 1 [file Table_1.docx]

**PubMed (1841)**

**((((((((((((((((((((((((((((((((((((((((((((((((((((((((((((((((((((((((((((((((((((((((((((((((((((((((((((((((((((((((((((((((((((((Fatty Acids, Omega-3) OR (N-3 Fatty Acid)) OR (Acid, N-3 Fatty)) OR (Fatty Acid, N-3)) OR (N 3 Fatty Acid)) OR (Omega-3 Fatty Acid)) OR (Acid, Omega-3 Fatty)) OR (Fatty Acid, Omega-3)) OR (Omega 3 Fatty Acid)) OR (Omega-3 Fatty Acids)) OR (n-3 Oil)) OR (n 3 Oil)) OR (Oil, n-3)) OR (n3 Oil)) OR (Oil, n3)) OR (n-3 Fatty Acids)) OR (n 3 Fatty Acids)) OR (n-3 Polyunsaturated Fatty Acid)) OR (n 3 Polyunsaturated Fatty Acid)) OR (n-3 PUFA)) OR (n 3 PUFA)) OR (PUFA, n-3)) OR (n3 Fatty Acid)) OR (Fatty Acid, n3)) OR (n3 PUFA)) OR (PUFA, n3)) OR (n3 Polyunsaturated Fatty Acid)) OR (n3 Oils)) OR (Omega 3 Fatty Acids)) OR (n-3 Oils)) OR (n 3 Oils)) OR (Fish Oils)) OR (Oils, Fish)) OR (Fish Oil)) OR (Oil, Fish)) OR (Fish Liver Oils)) OR (Liver Oils, Fish)) OR (Oils, Fish Liver)) OR (Olive Oil)) OR (Oil, Olive)) OR (Oils, Olive)) OR (Olive Oils)) OR (Soybean Oil)) OR (Oil, Soybean)) OR (Oils, Soybean)) OR (Soybean Oils)) OR (Soya Oil)) OR (Oil, Soya)) OR (Oils, Soya)) OR (Soya Oils)) OR (Soy Bean Oil)) OR (Oil, Soy Bean)) OR (Oils, Soy Bean)) OR (Soy Bean Oils)) OR (Fatty Acids, Unsaturated)) OR (Acids, Unsaturated Fatty)) OR (Unsaturated Fatty Acids)) OR (Unsaturated Fatty Acid)) OR (Acid, Unsaturated Fatty)) OR (Fatty Acid, Unsaturated)) OR (Polyunsaturated Fatty Acids)) OR (Acids, Polyunsaturated Fatty)) OR (Polyunsaturated Fatty Acid)) OR (Acid, Polyunsaturated Fatty)) OR (Fatty Acid, Polyunsaturated)) OR (Fatty Acids, Polyunsaturated)) OR (PUFA)) OR (PUFAs)) OR (Fatty Acids)) OR (Fatty Acid)) OR (Aliphatic Acids)) OR (Aliphatic Acid)) OR (Acid, Aliphatic)) OR (Fatty Acids, Esterified)) OR (Esterified Fatty Acids)) OR (Esterified Fatty Acid)) OR (Acid, Esterified Fatty)) OR (Fatty Acid, Esterified)) OR (Fatty Acids, Saturated)) OR (Saturated Fatty Acids)) OR (Saturated Fatty Acid)) OR (Acid, Saturated Fatty)) OR (Fatty Acid, Saturated)) OR (Fatty Acids, Essential)) OR (Acids, Essential Fatty)) OR (Essential Fatty Acids)) OR (EFA)) OR (EFAs)) OR (alpha-Linolenic Acid)) OR (alphalinolenic acid)) OR (ALA)) OR (ALAs)) OR (acid α-linolenic acid)) OR (alpha- linolenic acid)) OR (alpha Linolenic Acid)) OR (Docosahexaenoic Acids)) OR (Acids, Docosahexaenoic)) OR (Docosahexenoic Acids)) OR (Acids, Docosahexenoic)) OR (Docosahexaenoic Acid)) OR (Acid, Docosahexaenoic)) OR (DHA)) OR (DHAs)) OR (eicosapentenoic acid)) OR (EPA)) OR (Medium Chain Triglyceride*)) OR (MCT)) OR (Long Chain Triglyceride*)) OR (LCT)) OR (Omegaven)) OR (Smoflipid)) OR (Lipovenoes)) OR (Lipoplus)) OR (Intralipid)) OR (Lipofundin)) OR (Lipoven)) OR (Lipovenoess)) OR (Omegavenous)) OR (Clinoleic)) OR (Structolipid)) OR (Liposyn)) OR (omega 3)) OR (omega 6)) OR (w-3 fatty acid)) OR (n3)) OR (n-3)) OR (n6)) OR (krill oil)) OR (long chain PUFA)) OR (DPA)) OR (epas)) OR (lipid emulsion)) AND ((((((clinical trial) OR (randomized trial)) OR (randomized controlled trial)) OR (RCT)))))) AND ((((((((chronic) OR (persistent)) OR (intractable)) OR (long lasting)) OR (long-term)) OR (long term)) AND (((((((((((((((((((pain) OR (primary visceral pain)) OR (primary widespread pain)) OR (primary musculoskeletal pain)) OR (primary headache pain)) OR (primary orofacial pain)) OR (complex regional pain syndrome)) OR (CRPS)) OR (postsurgical pain)) OR (post-surgical pain)) OR (posttraumatic pain)) OR (post-traumatic pain)) OR (postoperative pain)) OR (post-operative pain)) OR (neuropathic pain)) OR (secondary headache pain)) OR (secondary orofacial pain)) OR (secondary visceral pain)) OR (secondary musculoskeletal pain))) OR ((((((((((chronic pain) OR (pain, intractable)) OR (facial neuralgia)) OR (facial neuralgia)) OR (fibromyalgia)) OR (fibromyalgia)) OR (pain, postoperative)) OR (neuralgia)) OR (neuralgia)) OR (complex regional pain syndromes)))**

**Web of science(1421)**

**#1 TS=(Fatty Acids, Omega-3 OR N-3 Fatty Acid OR Acid, N-3 Fatty OR Fatty Acid, N-3 OR N 3 Fatty Acid OR Omega-3 Fatty Acid OR Acid, Omega-3 Fatty OR Fatty Acid, Omega-3 OR Omega 3 Fatty Acid OR Omega-3 Fatty Acids OR n-3 Oil OR n 3 Oil OR Oil, n-3 OR n3 Oil OR Oil, n3 OR n-3 Fatty Acids OR n 3 Fatty Acids OR n-3 Polyunsaturated Fatty Acid OR n 3 Polyunsaturated Fatty Acid OR n-3 PUFA OR n 3 PUFA OR PUFA, n-3 OR n3 Fatty Acid OR Fatty Acid, n3 OR n3 PUFA OR PUFA, n3 OR n3 Polyunsaturated Fatty Acid OR n3 Oils OR Omega 3 Fatty Acids OR n-3 Oils OR n 3 Oils OR Fish Oils OR Oils, Fish OR Fish Oil OR Oil, Fish OR Fish Liver Oils OR Liver Oils, Fish OR Oils, Fish Liver OR Olive Oil OR Oil, Olive OR Oils, Olive OR Olive Oils OR Soybean Oil OR Oil, Soybean OR Oils, Soybean OR Soybean Oils OR Soya Oil OR Oil, Soya OR Oils, Soya OR Soya Oils OR Soy Bean Oil OR Oil, Soy Bean OR Oils, Soy Bean OR Soy Bean Oils OR Fatty Acids, Unsaturated OR Acids, Unsaturated Fatty OR Unsaturated Fatty Acids OR Unsaturated Fatty Acid OR Acid, Unsaturated Fatty OR Fatty Acid, Unsaturated OR Polyunsaturated Fatty Acids OR Acids, Polyunsaturated Fatty OR Polyunsaturated Fatty Acid OR Acid, Polyunsaturated Fatty OR Fatty Acid, Polyunsaturated OR Fatty Acids, Polyunsaturated OR PUFA OR PUFAs OR Fatty Acids OR Fatty Acid OR Aliphatic Acids OR Aliphatic Acid OR Acid, Aliphatic OR Fatty Acids, Esterified OR Esterified Fatty Acids OR Esterified Fatty Acid OR Acid, Esterified Fatty OR Fatty Acid, Esterified OR Fatty Acids, Saturated OR Saturated Fatty Acids OR Saturated Fatty Acid OR Acid, Saturated Fatty OR Fatty Acid, Saturated OR Fatty Acids, Essential OR Acids, Essential Fatty OR Essential Fatty Acids OR EFA OR EFAs OR alpha-Linolenic Acid OR alphalinolenic acid OR ALA OR ALAs OR acid α-linolenic acid OR alpha- linolenic acid OR alpha Linolenic Acid OR Docosahexaenoic Acids OR Acids, Docosahexaenoic OR Docosahexenoic Acids OR Acids, Docosahexenoic OR Docosahexaenoic Acid OR Acid, Docosahexaenoic OR DHA OR DHAs OR eicosapentenoic acid OR EPA OR Medium Chain Triglyceride* OR MCT OR Long Chain Triglyceride* OR LCT OR Omegaven OR Smoflipid OR Lipovenoes OR Lipoplus OR Intralipid OR Lipofundin OR Lipoven OR Lipovenoess OR Omegavenous OR Clinoleic OR Structolipid OR Liposyn OR omega 3 OR omega 6 OR w-3 fatty acid OR n3 OR n-3 OR n6 OR krill oil OR long chain PUFA OR DPA OR epas OR lipid emulsion)**

**#2 TS=(clinical trial OR randomized trial OR randomized controlled trial OR RCT)**

**#3 TS=(chronic OR persistent OR intractable OR long lasting OR long-term OR long term)) AND TS=(pain OR primary visceral pain OR primary widespread pain OR primary musculoskeletal pain OR primary headache pain OR primary orofacial pain)**

**#4 TS=(complex regional pain syndrome OR CRPS OR postsurgical pain OR post-surgical pain OR posttraumatic pain OR post-traumatic pain OR postoperative pain OR post-operative pain OR neuropathic pain OR secondary headache pain OR secondary orofacial pain OR secondary visceral pain OR secondary musculoskeletal pain OR chronic pain OR pain, intractable OR facial neuralgia OR facial neuralgia OR fibromyalgia OR fibromyalgia OR pain, postoperative OR neuralgia OR neuralgia OR complex regional pain syndromes)**

**#5 #3 OR #4**

**#6 #1 AND #2 AND #5**

**Embase（6277）**

**#1、'fatty acids, omega-3'/exp OR 'fatty acids, omega-3' OR 'n-3 fatty acid'/exp OR 'n-3 fatty acid' OR 'acid, n-3 fatty' OR 'fatty acid, n-3' OR 'n 3 fatty acid'/exp OR 'n 3 fatty acid' OR 'omega-3 fatty acid'/exp OR 'omega-3 fatty acid' OR 'acid, omega-3 fatty' OR 'fatty acid, omega-3' OR 'omega 3 fatty acid'/exp OR 'omega 3 fatty acid' OR 'omega-3 fatty acids' OR 'n-3 oil' OR 'n 3 oil' OR 'oil, n-3' OR 'n3 oil' OR 'oil, n3' OR 'n-3 fatty acids' OR 'n 3 fatty acids' OR 'n-3 polyunsaturated fatty acid'/exp OR 'n-3 polyunsaturated fatty acid' OR 'n 3 polyunsaturated fatty acid'/exp OR 'n 3 polyunsaturated fatty acid' OR 'n-3 pufa' OR 'n 3 pufa' OR 'pufa, n-3' OR 'n3 fatty acid' OR 'fatty acid, n3' OR 'n3 pufa' OR 'pufa, n3' OR 'n3 polyunsaturated fatty acid' OR 'n3 oils' OR 'omega 3 fatty acids' OR 'n-3 oils' OR 'n 3 oils' OR 'fish oils'/exp OR 'fish oils' OR 'oils, fish' OR 'fish oil'/exp OR 'fish oil' OR 'oil, fish' OR 'fish liver oils'/exp OR 'fish liver oils' OR 'liver oils, fish' OR 'oils, fish liver' OR 'olive oil'/exp OR 'olive oil' OR 'oil, olive'/exp OR 'oil, olive' OR 'oils, olive' OR 'olive oils' OR 'soybean oil'/exp OR 'soybean oil' OR 'oil, soybean' OR 'oils, soybean' OR 'soybean oils' OR 'soya oil'/exp OR 'soya oil' OR 'oil, soya' OR 'oils, soya' OR 'soya oils' OR 'soy bean oil'/exp OR 'soy bean oil' OR 'oil, soy bean' OR 'oils, soy bean' OR 'soy bean oils' OR 'fatty acids, unsaturated'/exp OR 'fatty acids, unsaturated' OR 'acids, unsaturated fatty' OR 'unsaturated fatty acids' OR 'unsaturated fatty acid'/exp OR 'unsaturated fatty acid' OR 'acid, unsaturated fatty' OR 'fatty acid, unsaturated'/exp OR 'fatty acid, unsaturated' OR 'polyunsaturated fatty acids' OR 'acids, polyunsaturated fatty' OR 'polyunsaturated fatty acid'/exp OR 'polyunsaturated fatty acid' OR 'acid, polyunsaturated fatty' OR 'fatty acid, polyunsaturated'/exp OR 'fatty acid, polyunsaturated' OR 'fatty acids, polyunsaturated' OR pufa OR pufas OR 'fatty acids'/exp OR 'fatty acids' OR 'fatty acid'/exp OR 'fatty acid' OR 'aliphatic acids' OR 'aliphatic acid'/exp OR 'aliphatic acid' OR 'acid, aliphatic' OR 'fatty acids, esterified' OR 'esterified fatty acids' OR 'esterified fatty acid'/exp OR 'esterified fatty acid' OR 'acid, esterified fatty' OR 'fatty acid, esterified' OR 'fatty acids, saturated' OR 'saturated fatty acids' OR 'saturated fatty acid'/exp OR 'saturated fatty acid' OR 'acid, saturated fatty' OR 'fatty acid, saturated'/exp OR 'fatty acid, saturated' OR 'fatty acids, essential'/exp OR 'fatty acids, essential' OR 'acids, essential fatty' OR 'essential fatty acids' OR efa OR efas OR 'alpha-linolenic acid'/exp OR 'alpha-linolenic acid' OR 'alphalinolenic acid' OR ala OR alas OR 'acid α-linolenic acid' OR 'alpha- linolenic acid'/exp OR 'alpha- linolenic acid' OR 'alpha linolenic acid'/exp OR 'alpha linolenic acid' OR 'docosahexaenoic acids'/exp OR 'docosahexaenoic acids' OR 'acids, docosahexaenoic' OR 'docosahexenoic acids' OR 'acids, docosahexenoic' OR 'docosahexaenoic acid'/exp OR 'docosahexaenoic acid' OR 'acid, docosahexaenoic' OR 'dha'/exp OR dha OR dhas OR 'eicosapentenoic acid'/exp OR 'eicosapentenoic acid' OR epa OR 'medium chain' OR 'mct'/exp OR mct OR 'long chain' OR lct OR 'omegaven'/exp OR omegaven OR 'smoflipid'/exp OR smoflipid OR 'lipovenoes'/exp OR lipovenoes OR 'lipoplus'/exp OR lipoplus OR 'intralipid'/exp OR intralipid OR 'lipofundin'/exp OR lipofundin OR 'lipoven'/exp OR lipoven OR lipovenoess OR omegavenous OR 'clinoleic'/exp OR clinoleic OR 'structolipid'/exp OR structolipid OR 'liposyn'/exp OR liposyn OR 'omega 3'/exp OR 'omega 3' OR 'omega 6' OR 'w-3 fatty acid' OR n3 OR 'n 3'/exp OR 'n 3' OR n6 OR 'krill oil'/exp OR 'krill oil' OR 'long chain pufa' OR dpa OR epas OR 'lipid emulsion'/exp OR 'lipid emulsion'**

**#2 'chronic primary visceral pain' OR 'chronic primary widespread pain' OR 'chronic primary musculoskeletal pain' OR 'chronic primary headache pain' OR 'chronic primary orofacial pain' OR 'persistent pain'/exp OR 'persistent pain' OR 'persistent primary visceral pain' OR 'persistent primary widespread pain' OR 'persistent primary musculoskeletal pain' OR 'persistent primary headache pain' OR 'persistent primary orofacial pain' OR 'intractable pain'/exp OR 'intractable pain' OR 'intractable primary visceral pain' OR 'intractable primary widespread pain' OR 'intractable primary musculoskeletal pain' OR 'intractable primary headache pain' OR 'intractable primary orofacial pain' OR 'long lasting pain' OR 'long lasting primary visceral pain' OR 'long lasting primary widespread pain' OR 'long lasting primary musculoskeletal pain' OR 'long lasting primary headache pain' OR 'long lasting primary orofacial pain' OR 'long-term pain' OR 'long-term primary visceral pain' OR 'long-term primary widespread pain' OR 'long-term primary musculoskeletal pain' OR 'long-term primary headache pain' OR 'long-term primary orofacial pain' OR 'long term pain' OR 'long term primary visceral pain' OR 'long term primary widespread pain' OR 'long term primary musculoskeletal pain' OR 'long term primary headache pain' OR 'long term primary orofacial pain' OR crps OR 'postsurgical pain'/exp OR 'postsurgical pain' OR 'post-surgical pain'/exp OR 'post-surgical pain' OR 'posttraumatic pain'/exp OR 'posttraumatic pain' OR 'post-traumatic pain'/exp OR 'post-traumatic pain' OR 'postoperative pain'/exp OR 'postoperative pain' OR 'post-operative pain'/exp OR 'post-operative pain' OR 'neuropathic pain'/exp OR 'neuropathic pain' OR 'secondary headache pain' OR 'secondary orofacial pain' OR 'secondary visceral pain' OR 'secondary musculoskeletal pain' OR 'chronic pain'/exp OR 'chronic pain' OR 'pain, intractable'/exp OR 'pain, intractable' OR 'facial neuralgia'/exp OR 'facial neuralgia' OR 'fibromyalgia'/exp OR fibromyalgia OR 'pain, postoperative'/exp OR 'pain, postoperative' OR 'neuralgia'/exp OR neuralgia OR 'complex regional pain syndrome'/exp OR 'complex regional pain syndrome'**

**#3 'clinical trial'/exp OR 'clinical trial' OR 'randomized trial' OR 'randomized controlled trial'/exp OR 'randomized controlled trial' OR rct**

**#4 #1 and #2 and #3 and 'article'/it**

**Cochrane library（985）**


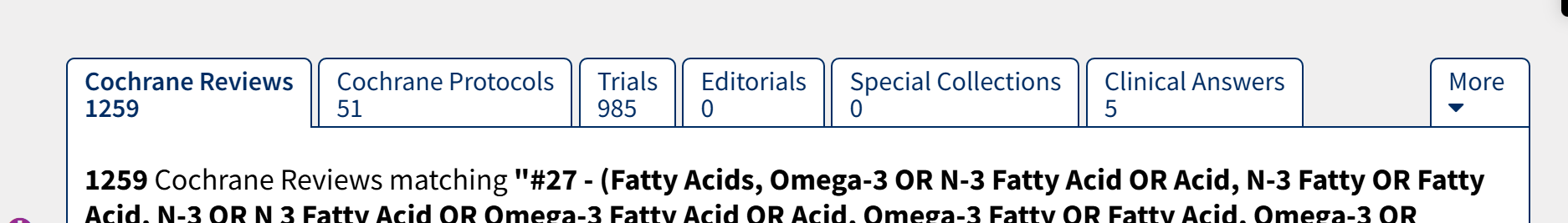


**(Fatty Acids, Omega-3 OR N-3 Fatty Acid OR Acid, N-3 Fatty OR Fatty Acid, N-3 OR N 3 Fatty Acid OR Omega-3 Fatty Acid OR Acid, Omega-3 Fatty OR Fatty Acid, Omega-3 OR Omega 3 Fatty Acid OR Omega-3 Fatty Acids OR n-3 Oil OR n 3 Oil OR Oil, n-3 OR n3 Oil OR Oil, n3 OR n-3 Fatty Acids OR n 3 Fatty Acids OR n-3 Polyunsaturated Fatty Acid OR n 3 Polyunsaturated Fatty Acid OR n-3 PUFA OR n 3 PUFA OR PUFA, n-3 OR n3 Fatty Acid OR Fatty Acid, n3 OR n3 PUFA OR PUFA, n3 OR n3 Polyunsaturated Fatty Acid OR n3 Oils OR Omega 3 Fatty Acids OR n-3 Oils OR n 3 Oils OR Fish Oils OR Oils, Fish OR Fish Oil OR Oil, Fish OR Fish Liver Oils OR Liver Oils, Fish OR Oils, Fish Liver OR Olive Oil OR Oil, Olive OR Oils, Olive OR Olive Oils OR Soybean Oil OR Oil, Soybean OR Oils, Soybean OR Soybean Oils OR Soya Oil OR Oil, Soya OR Oils, Soya OR Soya Oils OR Soy Bean Oil OR Oil, Soy Bean OR Oils, Soy Bean OR Soy Bean Oils OR Fatty Acids, Unsaturated OR Acids, Unsaturated Fatty OR Unsaturated Fatty Acids OR Unsaturated Fatty Acid OR Acid, Unsaturated Fatty OR Fatty Acid, Unsaturated OR Polyunsaturated Fatty Acids OR Acids, Polyunsaturated Fatty OR Polyunsaturated Fatty Acid OR Acid, Polyunsaturated Fatty OR Fatty Acid, Polyunsaturated OR Fatty Acids, Polyunsaturated OR PUFA OR PUFAs OR Fatty Acids OR Fatty Acid OR Aliphatic Acids OR Aliphatic Acid OR Acid, Aliphatic OR Fatty Acids, Esterified OR Esterified Fatty Acids OR Esterified Fatty Acid OR Acid, Esterified Fatty OR Fatty Acid, Esterified OR Fatty Acids, Saturated OR Saturated Fatty Acids OR Saturated Fatty Acid OR Acid, Saturated Fatty OR Fatty Acid, Saturated OR Fatty Acids, Essential OR Acids, Essential Fatty OR Essential Fatty Acids OR EFA OR EFAs OR alpha-Linolenic Acid OR alphalinolenic acid OR ALA OR ALAs OR acid α-linolenic acid OR alpha-linolenic acid OR alpha Linolenic Acid OR Docosahexaenoic Acids OR Acids, Docosahexaenoic OR Docosahexenoic Acids OR Acids, Docosahexenoic OR Docosahexaenoic Acid OR Acid, Docosahexaenoic OR DHA OR DHAs OR eicosapentenoic acid OR EPA OR Medium Chain Triglyceride* OR MCT OR Long Chain Triglyceride* OR LCT OR Omegaven OR Smoflipid OR Lipovenoes OR Lipoplus OR Intralipid OR Lipofundin OR Lipoven OR Lipovenoess OR Omegavenous OR Clinoleic OR Structolipid OR Liposyn OR omega 3 OR omega 6 OR w-3 fatty acid OR n3 OR n-3 OR n6 OR krill oil OR long chain PUFA OR DPA OR epas OR lipid emulsion) AND (chronic pain OR chronic primary visceral pain OR chronic primary widespread pain OR chronic primary musculoskeletal pain OR chronic primary headache pain OR chronic primary orofacial pain OR persistent pain OR persistent primary visceral pain OR persistent primary widespread pain OR persistent primary musculoskeletal pain OR persistent primary headache pain OR persistent primary orofacial pain OR intractable pain OR intractable primary visceral pain OR intractable primary widespread pain OR intractable primary musculoskeletal pain OR intractable primary headache pain OR intractable primary orofacial pain OR long lasting pain OR long lasting primary visceral pain OR long lasting primary widespread pain OR long lasting primary musculoskeletal pain OR long lasting primary headache pain OR long lasting primary orofacial pain OR long-term pain OR long-term primary visceral pain OR long-term primary widespread pain OR long-term primary musculoskeletal pain OR long-term primary headache pain OR long-term primary orofacial pain OR long term pain OR long term primary visceral pain OR long term primary widespread pain OR long term primary musculoskeletal pain OR long term primary headache pain OR long term primary orofacial pain OR complex regional pain syndrome OR CRPS OR postsurgical pain OR post-surgical pain OR posttraumatic pain OR post-traumatic pain OR postoperative pain OR post-operative pain OR neuropathic pain OR secondary headache pain OR secondary orofacial pain OR secondary visceral pain OR secondary musculoskeletal pain OR chronic pain OR pain, intractable OR facial neuralgia OR facial neuralgia OR fibromyalgia OR fibromyalgia OR pain, postoperative OR neuralgia OR neuralgia OR complex regional pain syndrome) AND (clinical trial OR randomized trial OR randomized controlled trial OR RCT)**
