# Supplementary material for: Effects of omega-3 fatty acids on chronic pain: a systematic review and meta-analysis
Source: Front Med (Lausanne). 2025 Nov 5;12:1654661. doi: 10.3389/fmed.2025.1654661 (PMC12627051; doi:10.3389/fmed.2025.1654661)
Supplement: Supplementary file 3 [file Data_Sheet_1.docx]

**
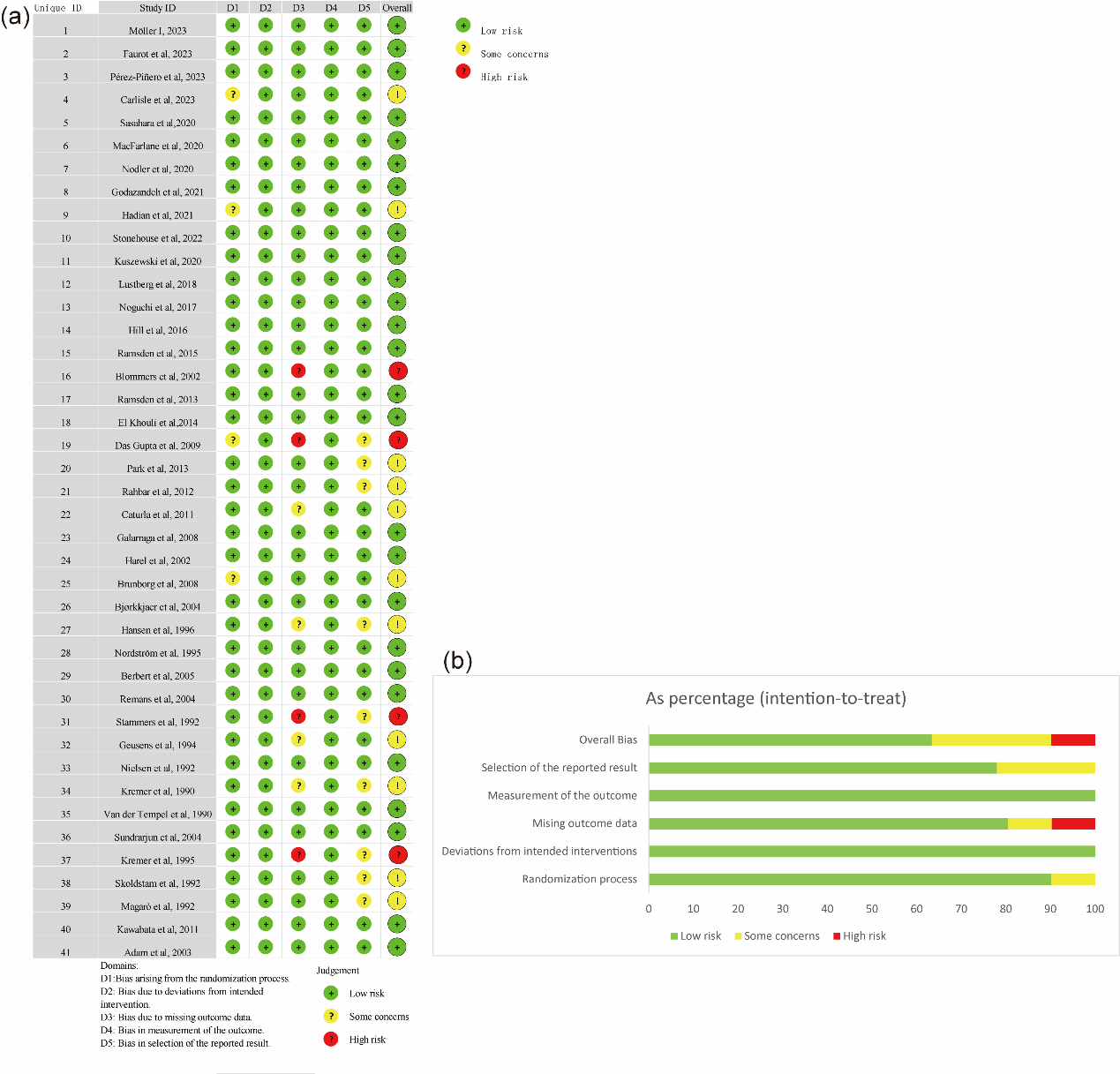
Figure S1** (a)Risk-of-bias summary of studies examining the effects of the omega-3 fatty acids; (b) Risk-of-bias graph of studies examining the effects of the omega-3 fatty acid


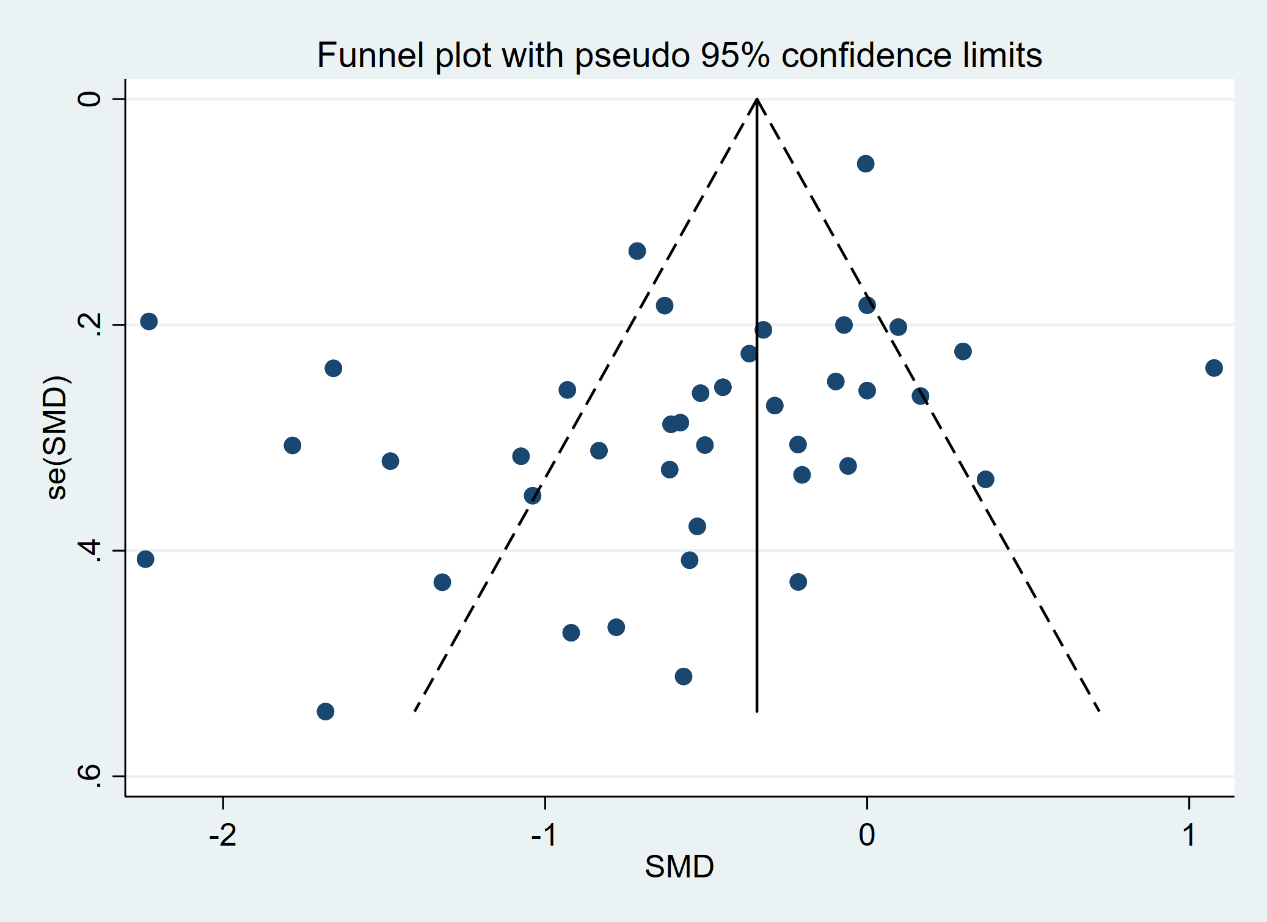
**Figure S2.** Funnel plot for assessing publication bias across studies evaluating the effect of omega-3 fatty acid supplementation on chronic pain.


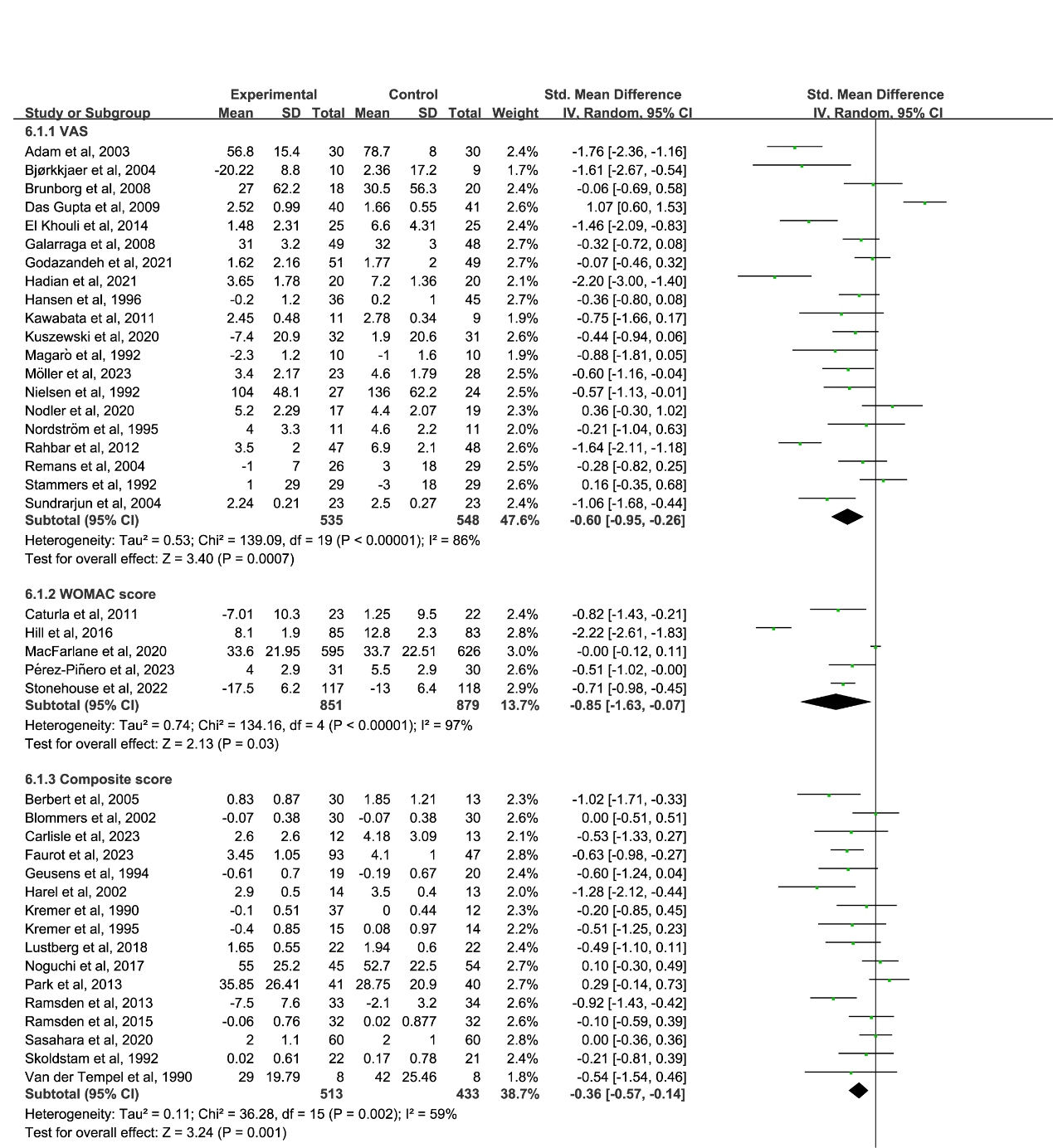


**Figure S3.** Forest plot of the effect of omega-3 fatty acid supplementation on chronic pain stratified by pain assessment tool.

**
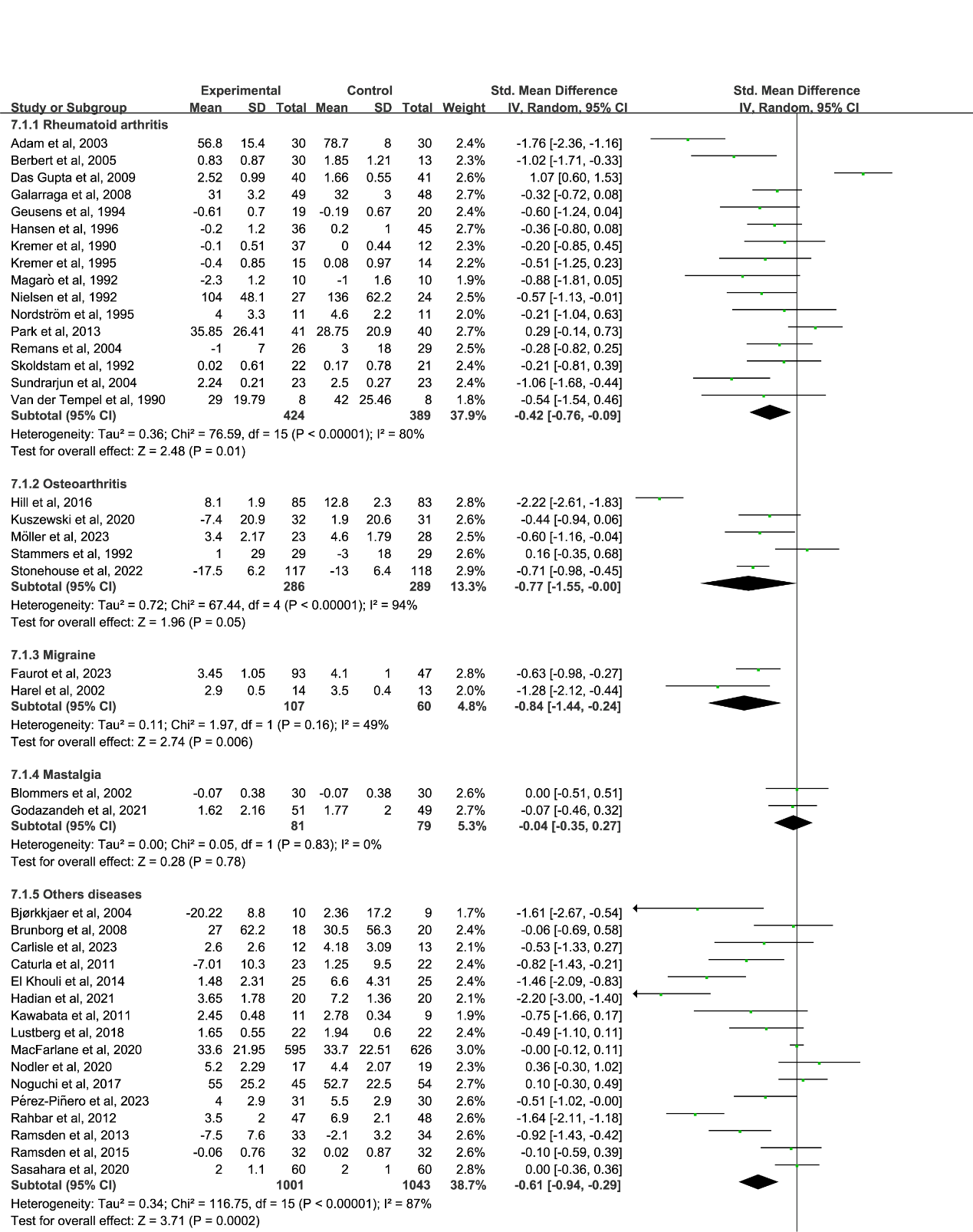
Figure S4.** Forest plot of the effect of omega-3 fatty acid supplementation on chronic pain stratified by disease type.

**
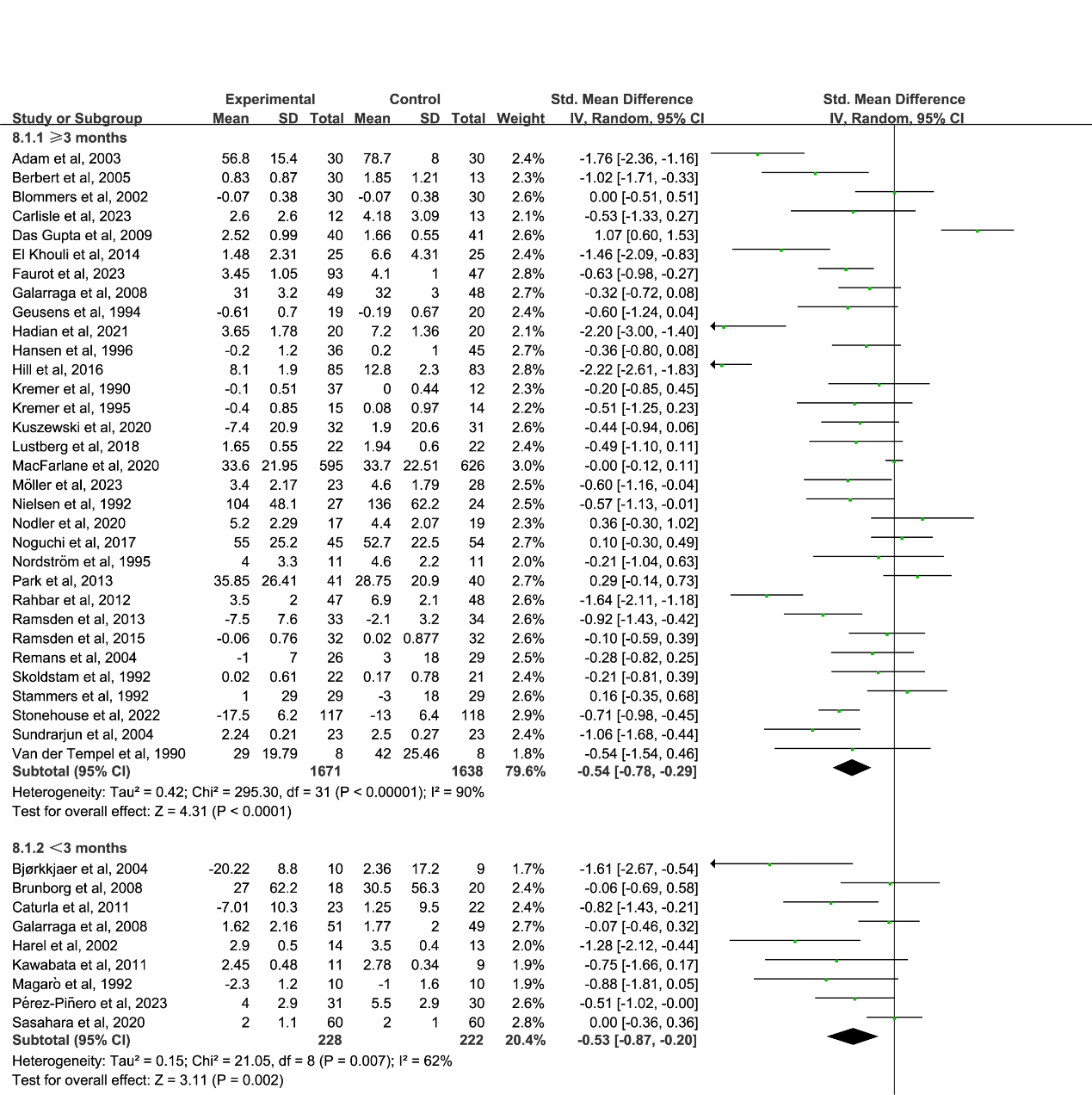
Figure S5.** Forest plot of the effect of omega-3 fatty acid supplementation on chronic pain stratified by intervention duration.

**
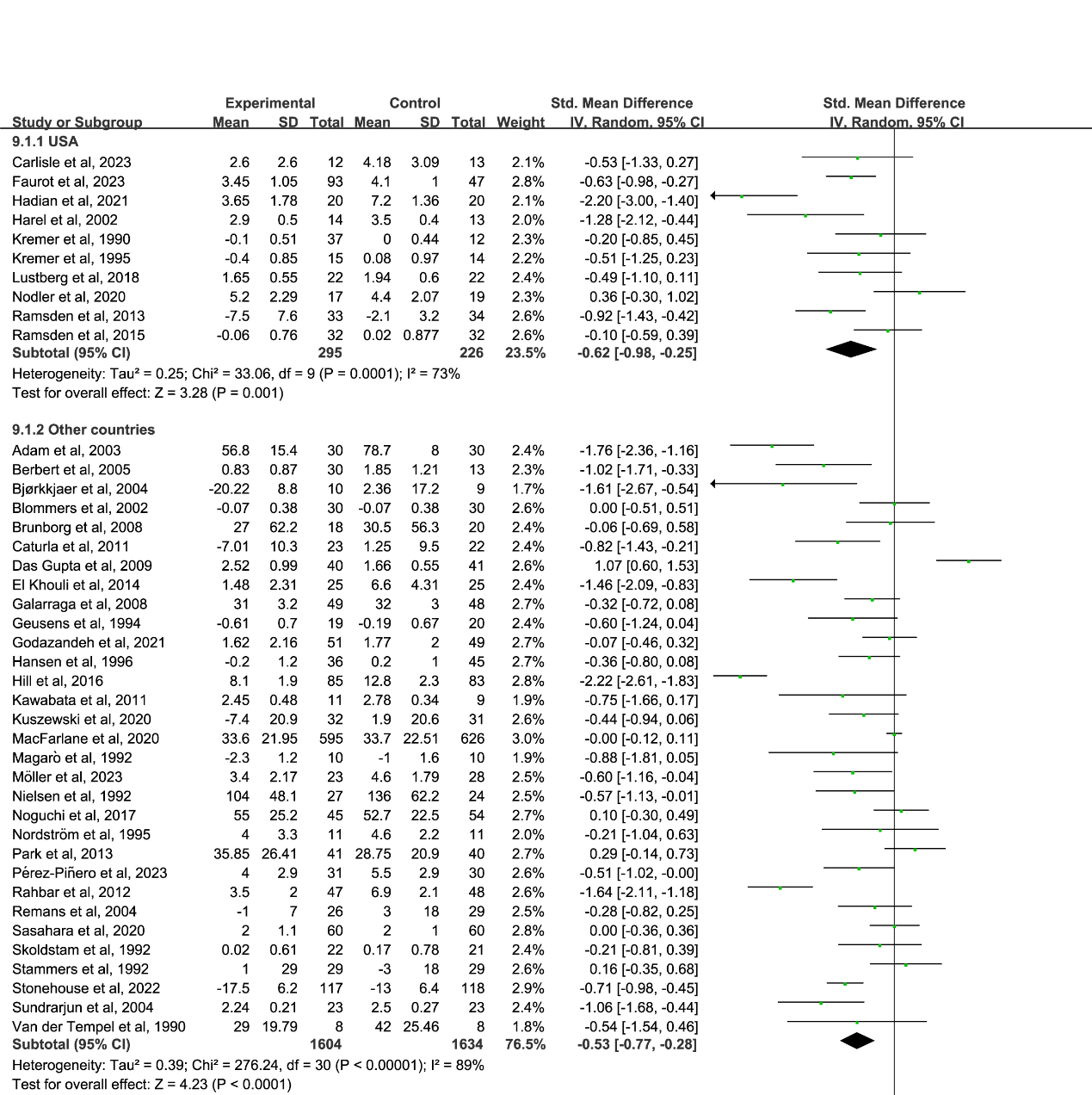
**

**Figure S6.** Forest plot of the effect of omega-3 fatty acid supplementation on chronic pain stratified by geographic location.

**
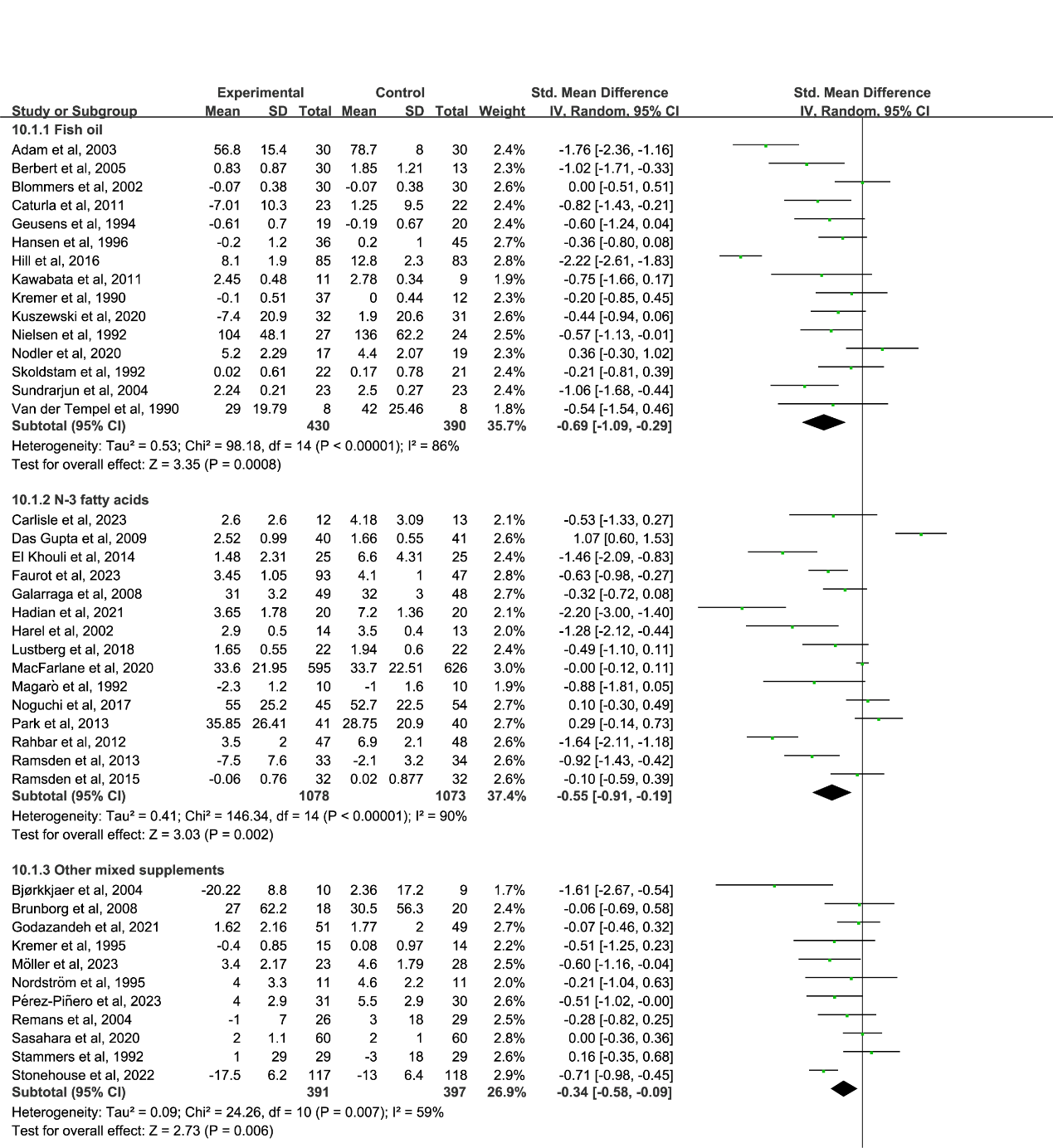
Figure S7.** Forest plot of the effect of omega-3 fatty acid supplementation on chronic pain stratified by type of fatty acid supplement.

**
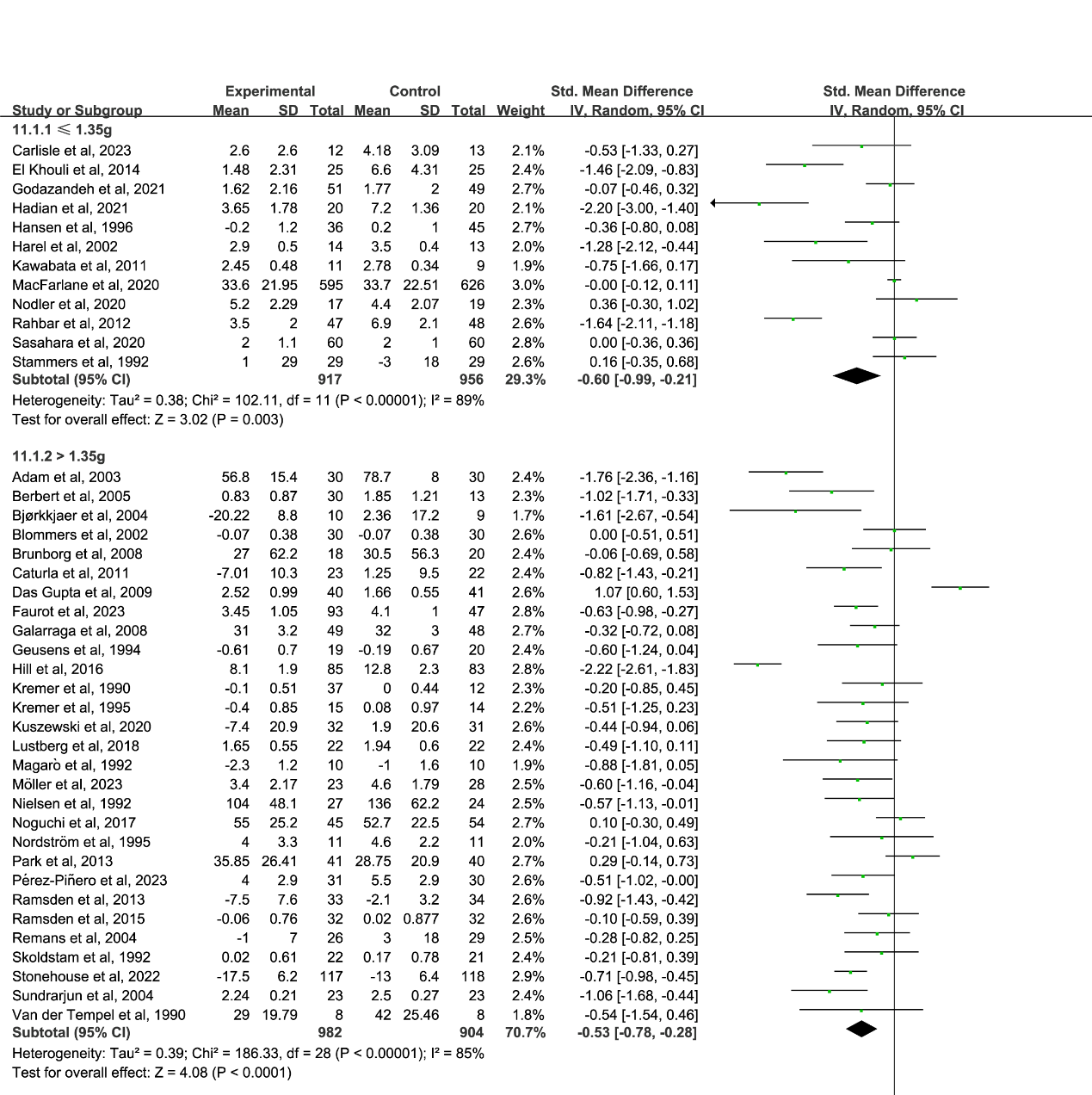
**

**Figure S8**. Forest plot of the effect of omega-3 fatty acid supplementation on chronic pain stratified by daily dosage.

**
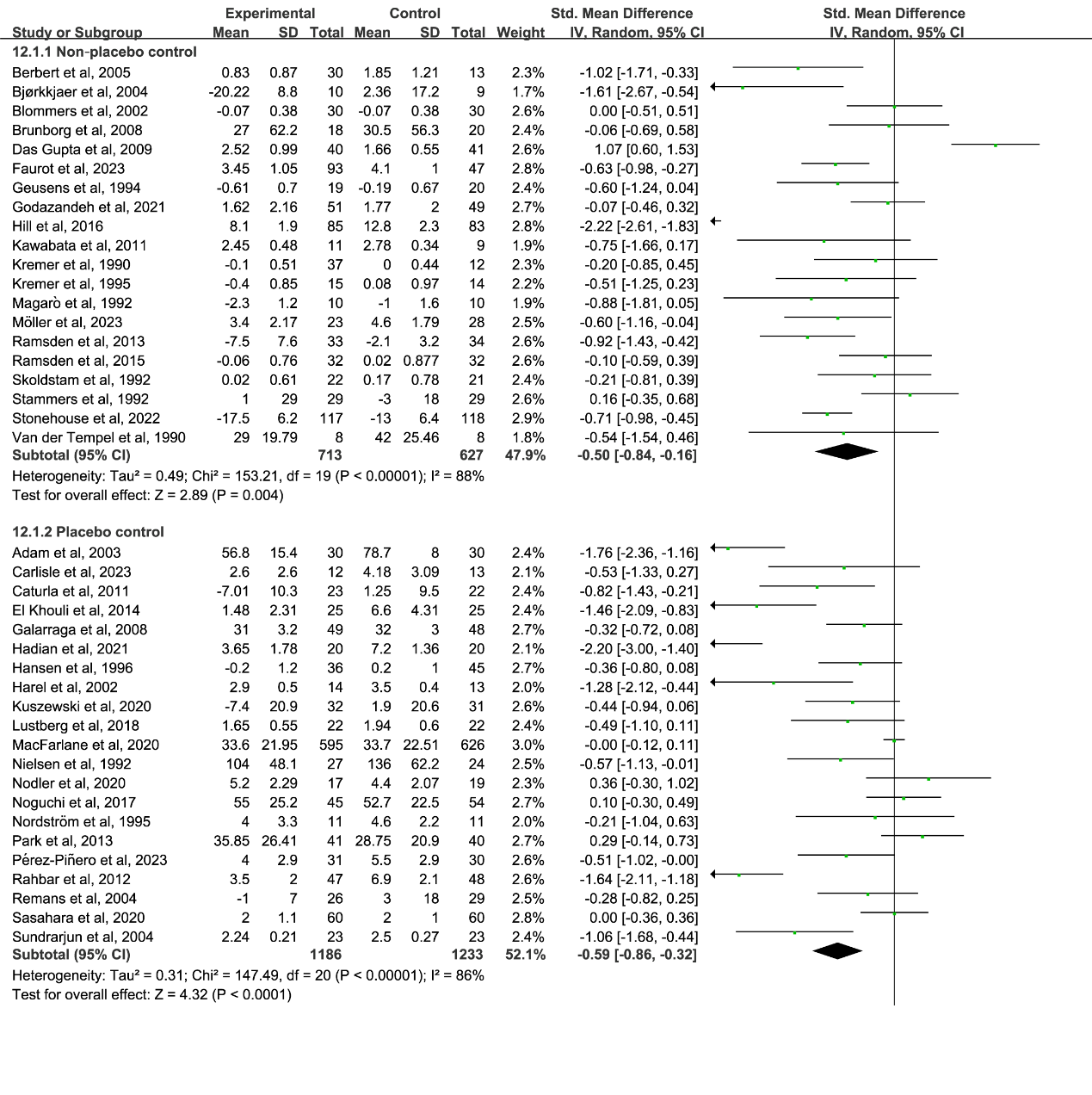
Figure S9.** Forest plot of the effect of omega-3 fatty acid supplementation on chronic pain stratified by control type.

**Figure S10.** Filled funnel plot from trim-and-fill analysis. Open circles plot the observed study-level effect sizes (θ) against their standard errors (SE). The trim-and-fill algorithm iteratively removed the most asymmetric positive studies, then mirrored and “filled” the presumed missing counterparts (filled squares) to restore symmetry about the pooled effect.
